# Elucidation of the RamA Regulon in *Klebsiella pneumoniae* Reveals a Role in LPS Regulation

**DOI:** 10.1371/journal.ppat.1004627

**Published:** 2015-01-29

**Authors:** Shyamasree De Majumdar, Jing Yu, Maria Fookes, Sean P. McAteer, Enrique Llobet, Sarah Finn, Shaun Spence, Avril Monaghan, Adrien Kissenpfennig, Rebecca J. Ingram, José Bengoechea, David L. Gally, Séamus Fanning, Joseph S. Elborn, Thamarai Schneiders

**Affiliations:** 1 Centre for Infection and Immunity, Belfast, United Kingdom; 2 Division of Pathway and Infection Medicine, Edinburgh, United Kingdom; 3 Wellcome Trust Sanger Institute, Wellcome Trust Genome Campus, Hinxton, Cambridge, United Kingdom; 4 Division of Immunity and Infection, The Roslin Institute and R(D)SVS, The University of Edinburgh, Easter Bush, Midlothian, United Kingdom; 5 Laboratory Microbial Pathogenesis, Fundació d’Investigació Sanitària de les Illes Balears (FISIB) Recinto Hospital Joan March, Bunyola, Spain; 6 UCD Centre for Molecular Innovation and Drug Discovery, School of Public Health, Physiotherapy & Population Science, University College Dublin, Dublin, Ireland; Northwestern University, UNITED STATES

## Abstract

*Klebsiella pneumoniae* is a significant human pathogen, in part due to high rates of multidrug resistance. RamA is an intrinsic regulator in *K. pneumoniae* established to be important for the bacterial response to antimicrobial challenge; however, little is known about its possible wider regulatory role in this organism during infection. In this work, we demonstrate that RamA is a global transcriptional regulator that significantly perturbs the transcriptional landscape of *K. pneumoniae*, resulting in altered microbe-drug or microbe-host response. This is largely due to the direct regulation of 68 genes associated with a myriad of cellular functions. Importantly, RamA directly binds and activates the *lpxC*, *lpxL-2* and *lpxO* genes associated with lipid A biosynthesis, thus resulting in modifications within the lipid A moiety of the lipopolysaccharide. RamA-mediated alterations decrease susceptibility to colistin E, polymyxin B and human cationic antimicrobial peptide LL-37. Increased RamA levels reduce *K. pneumoniae* adhesion and uptake into macrophages, which is supported by *in vivo* infection studies, that demonstrate increased systemic dissemination of ramA overexpressing *K. pneumoniae*. These data establish that RamA-mediated regulation directly perturbs microbial surface properties, including lipid A biosynthesis, which facilitate evasion from the innate host response. This highlights RamA as a global regulator that confers pathoadaptive phenotypes with implications for our understanding of the pathogenesis of *Enterobacter*, *Salmonella* and *Citrobacter* spp. that express orthologous RamA proteins.

## Author Summary

Bacteria can rapidly evolve under antibiotic pressure to develop resistance, which occurs when target genes mutate, or when resistance-encoding genes are transferred. Alternatively, microbes can simply alter the levels of intrinsic proteins that allow the organism to “buy” time to resist antibiotic pressure. *Klebsiella pneumoniae* is a pathogen that causes significant blood stream or respiratory infections, but more importantly is a bacterium that is increasingly being reported as multidrug resistant. Our data demonstrate that RamA can trigger changes on the bacterial surface that allow *Klebsiella* to survive both antibiotic challenge, degradation by host immune peptides and resist phagocytosis. We demonstrate that the molecular basis of increased survival of *ramA* overexpressing *K. pneumoniae*, against host-derived factors is associated with RamA-driven alterations of the lipid A moiety of *Klebsiella* LPS. This modification is likely to be linked to *Klebsiella’s* ability to resist the host response so that it remains undetected by the immune system. The relevance of our work extends beyond RamA in *Klebsiella* as other pathogens such as *Enterobacter* spp and *Salmonella* spp. also produce this protein. Thus our overarching conclusion is that the intrinsic regulator, RamA perturbs host-microbe and microbe-drug interactions.

## Introduction

The microbial response to antimicrobial challenge is multifactorial and can be conferred by a combination of extrinsic or intrinsic mechanisms. Those intrinsic mechanisms that confer pleiotropic phenotypes can provide a “stepping stone” to surmounting both the host or drug response. Intrinsic proteins such as the AraC-transcriptional proteins e.g. MarA [1], SoxS [2], Rob [3], RamA [4] and RarA [5], directly regulate genes linked to microbial permeability barriers which results in reduced susceptibility [6] to multiple antibiotic classes. The perturbation of the permeability barrier is identified as a critical step in the development and emergence of higher levels of resistance [7].

The regulatory proteins, typified by the MarA protein, are unique, as unlike other members of the AraC family, these proteins bind DNA as monomers [8], interact with RNA polymerase via a process of pre-recruitment [9] and generally confer reduced antimicrobial susceptibility [10]. Microarray analyses has highlighted the wider effects of increased MarA [1], SoxS [2], RamA [4, 11] and RarA [5] levels in modulating gene expression particularly of those genes linked to virulence. This is further supported by studies reporting that either the inhibition or deletion of these regulators [12] can impair the ability of *E. coli* to colonise and cause infection *in vivo* [13]. Taken together, it is evident that these AraC proteins can confer bifunctional phenotypes of reduced drug susceptibility and increased virulence, which facilitate pathogen survival. These findings firstly, underscore the relative importance of these factors in microbial survival and secondly, provide a rationale for the development of “Anti-virulence-type” inhibitors against these transcription proteins.

**Figure 1:**
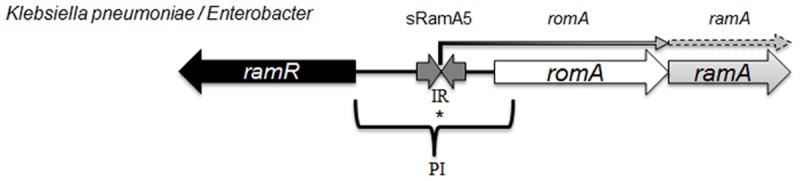
Organisation of the *ram* locus in *Klebsiella pneumoniae / Enterobacter* spp. In *K. pneumoniae romA* and *ramA* are usually co-transcribed from the TSS depicted with *, under the control of the PI promoter. RamR can bind the inverted repeat (atgagtgn_6_cactcat), which in turn represses the transcription of both *romA* and *ramA*. Our analysis shows that the small regulatory RNA, sRamA5 and *romA* in *Klebsiella pneumoniae* share the TSS (depicted with *).

The *ramA* gene which encodes the RamA protein is found in *Klebsiella*, *Enterobacter* [14], *Salmonella* [15] and *Citrobacter* spp [16] where the genetic organisation of the *ram* locus is conserved in most organisms, with the exception of *Salmonella enterica* serovar Typhimurium (Fig. 1) which lacks *romA*, a putative metallo-beta-lactamase gene. The levels of both the *romA-ramA* genes are repressed at the transcriptional level by the TetR-type family regulator RamR, encoded by the *ramR* gene, which is divergently transcribed from the *romA-ramA* operon. In both *Klebsiella and Salmonella*, an increase in *ramA* expression can be mediated by inactivating mutations [16–18] or ligand mediated interactions [19] with the cognate repressor, RamR which binds to a highly conserved inverted repeat (atgagtgn_6_cactcat) [20] overlapping the promoter region of the *romAramA* operon (Fig. 1). Mutations within the *ramR* gene in *K. pneumoniae* resulting in *ramA* overexpression were initially reported as a result of tigecycline exposure [17, 21]. However, previous work evaluating clinical isolates that pre-date the use of tigecycline demonstrate that *ramA* overexpressing strains were already present within the nosocomial population of *K. pneumoniae*, suggesting a broader role for RamA mediated overexpression in antibiotic resistance [16]. Interestingly, studies evaluating the prevalence of *ramA*-mediated overexpression in clinical isolates of *K. pneumoniae* and *Salmonella* spp. indicate that these bacteria are more likely to overexpress *ramA* than *marA* or *soxS*, suggesting that elevated *ramA* levels may be more relevant to the development of antibiotic resistance in these organisms.

Several studies [4, 11] have addressed the scope of the RamA regulon in *Salmonella enterica* serovar Typhimurium using microarray profiling. These studies demonstrate that *ramA* overexpression results in reduced antimicrobial susceptibility due to the differential regulation of *acrAB* and *micF* genes, which consequently decrease OmpF levels. One study [4] suggests that genes linked to the Salmonella Pathogenicity Island (SPI-2) are also differentially expressed, leading to the initial observation that RamA may impact on *Salmonella*-specific virulence attributes. However this link was not corroborated in subsequent *in vivo* experiments. In *K. pneumoniae*, the wider impact of RamA-mediated regulation is not known. Despite the apparent similarities in genome structure, the microbial lifestyles of both *K. pneumoniae* [22] and *Salmonella* spp. differ. Importantly, the increasing multidrug resistance in *Klebsiella* spp. demands a thorough understanding of factors within this genus that contribute to the intrinsic microbial ‘resistome’ and survival under selective (host or drug) pressure. Therefore to define the broad effects of RamA-mediated expression on microbe-host and microbe-drug phenotypes we carried out transcriptome profiling using directional RNAseq with the wild type strain *K. pneumoniae* Ecl8 [23] and its isogenic derivatives Ecl8Δ*ramA* and Ecl8Δ*ramR*. Our key findings show the scope of RamA-mediated regulation significantly alters the transcriptional landscape of *K. pneumoniae*. This occurs by directly modulating the expression of different genes notably those associated with antimicrobial resistance and host-microbe interactions thereby resulting in the emergence of a less antibiotic susceptible and more virulent *K. pneumoniae*.

## Results

### Regulation of the *ram* Locus

**The *ram* locus encodes a sRNA to maintain basal levels of *ramA* expression**. RamR functions as the primary repressor of both *romA-ramA* expression in *K. pneumoniae* by binding the palindromic repeats of the IR element which flanks the TSS for *romA* at position -64T. *ramR*, itself, has two transcriptional start sites, located at the -83T and -167A positions where expression analyses using GFP fusions suggest that the primary promoter region for *ramR* transcription is located at the -83T start site (S1 and S2 Figs.). This site is also repressed 5-fold more than the vector only control by *ramR* in trans indicating that like other TetR-type regulators, RamR expression is autoregulated (S2 Fig.).

**Figure 2:**
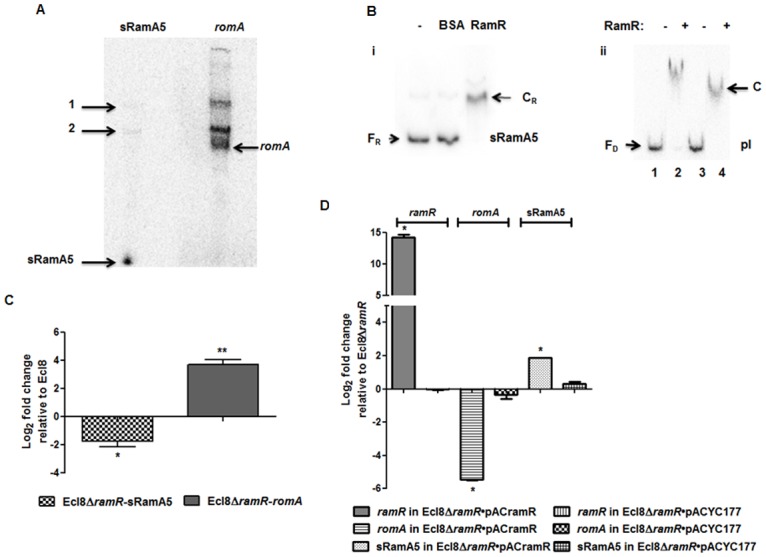
A: Northern blot analysis of sRamA5. 15 μg of total RNA extracted from Ecl8/pGEMTpI+*romA* were loaded into wells. The blots were either incubated with the ^32^P-end labelled sRamA5 specific DNA probe or *romA* specific DNA probe. The bands pointed as 1 and 2 are primary transcripts for both the RNA codes for sRamA5 and *romA*. The band referred to as sRamA5 is specifically detected by the sRamA5 DNA probe, sized at around 60 nucleotides; the band referred to as *romA* was specifically detected by the *romA* DNA probe. **B:** EMSA of RamR-sRamA5 or RamR-PI interaction in the presence of sRamA5. (i). RamR-sRamA5 interaction. The concentrations of sRamA5 and RamR were 40 nM and 1 μM respectively. (ii). RamR-pI interaction in the absence/presence of sRamA5. Radioactive labeled pI was 2 nM from lane 1 to 4. RamR’s concentrations from lane 1 to 4 were: 0, 2, 0, 2 μM. Cold sRamA5’s concentrations from lane 1 to 4 were: 0, 0, 1, 1 μM. F_R_ = free RNA, C_R_ = RNA-protein complex, F_D_ = free DNA, C = RNA-DNA-protein complex. **C:** qPCR for the level of *romA* and sRamA5’s transcriptions in Ecl8Δ*ramR*. qPCR using LNA probe for determining the levels of sRamA5 transcription in Ecl8 and Ecl8Δ*ramR*. Despite sharing the same TSS, the transcript levels of sRamA5 are not linked to *romA* levels, thereby reducing the likelihood of sRamA5 being a 5’ untranslated region of *romA*. The log_2_ fold changes in Ecl8Δ*ramR* displayed in the bar chart are relative to their transcript levels in Ecl8. One-way ANOVA analyses (P<0.001) were performed to demonstrate statistical significance. **D:** qPCR assay for the level of *ramR*, *romA* and sRamA5 in Ecl8Δ*ramR* pACYC*ramR* and Ecl8Δ*ramR* pACYC177. The log_2_ fold changes in the two strains displayed in the bar chart are relative to their transcript levels in Ecl8Δ*ramR*. sRamA5 levels are elevated in the presence of *ramR*, implying that RamR could stabilise the sRamA5 transcript. One-way ANOVA analyses (P<0.001) were performed to demonstrate statistical significance.

Previous work in *Salmonella* has shown that the regulatory RNA, StyR3, can control expression at the *ram* locus [24]. Given the expansive role of *ramA* in gene regulation, we sought to determine whether the *K. pneumoniae* ortholog of StyR3, denoted as sRamA5, would function as co-regulator of *ramA* expression in *K. pneumoniae* to promote basal *ramA* levels. The lack of similarity within the intergenic regions located between the *ramR* and *romA-ramA* genes or *ramR* and *ramA* genes in *K. pneumoniae* and *Salmonella* spp. respectively, excluded the possibility of using sequence analyses to identify the StyR3 ortholog. Direct northern blot analyses of RNA derived from *K. pneumoniae* strain Ecl8 and its derivatives did not produce a detectable signal for the putative regulatory RNA, sRamA5. Thus in order to demonstrate the presence of sRamA5, we cloned the entire intergenic region flanked by the *ramR* and *romA* genes and the partial *romA* open reading frame into the TA cloning vector pGEMTeasy to generate pGEMsRamA5. Northern blot analyses derived from the expression of sRamA5 encoded on pGEMsRamA5, using gene specific probes for sRamA5 and *romA* ORF, demonstrate the presence of sRamA5 (~ 60nt) (shown in Fig. 2A). Notably, the sRamA5 specific probe also detected a further two RNA molecules (Fig. 2A, arrowed bands 1 and 2). These fragments, detected by both the sRamA5 and *romA* specific probe, possibly represent primary transcripts initiated from the common start site as determined by 5’ RACE analyses for sRamA5 and *romA* (S1 Fig.). As expected the *romA* specific probe did not detect the 60nt sRamA5 molecule (Fig. 2A). Thus we surmise that sRamA5 and *romA* are co-transcribed into a primary RNA molecule, which undergoes further processing prior to excision proximal to the start of the *romA* gene, thereby producing sRamA5.

As a classical TetR-family protein, RamR-mediated repression of the *romA-ramA* locus is likely to be perturbed through ligand-mediated interactions; therefore we hypothesized that to function as a co-regulator of *romA-ramA* expression RamR would interact with sRamA5. RNA-EMSA (S1 Text) analyses demonstrate that RamR and sRamA5 form a complex, suggesting direct interaction of the RNA (sRamA5) with RamR (Fig. 2B). In order to ascertain whether the interaction of sRamA5 and RamR is attributable to the presence of the highly conserved IR sequence in the *ramR-romA* inetrgenic region (ATGAGTGcgtactCACTCAT) and thus, act as a competitor for RamR-pI binding, we performed EMSA analyses using the pI promoter, sRamA5 and RamR. Our results show a reduction in affinity of RamR to sRamA5 in the presence of excess pI promoter (Fig. 2B). In contrast, competition experiments with excess sRamA5 show no perturbation of the pI+RamR interaction, suggesting that RamR has a higher affinity for the pI promoter compared to sRamA5 (Fig. 2B).

Simultaneous qPCR measurements utilizing an LNA probe to assess sRamA5 levels demonstrate firstly, that the transcription levels of sRamA5 and *romA* are not linked as sRamA5 levels are decreased in contrast to elevated *romA* levels (Fig. 2C). This suggests that despite being transcribed from the same TSS, sRamA5 and *romA* are likely subject to different rates of degradation. Secondly, the stability of sRamA5 may be dependent on the presence of a functional RamR. In order to investigate the requirement for a functional RamR in sRamA5 stability, we determined both the *romA* and sRamA5 levels in Ecl8Δ*ramR* before and after complementation with *ramR* expressed in *trans*. As expected, our results show that the level of *romA* transcription was reduced (∼ 30-fold) in Ecl8Δ*ramR*/pAC*ramR* compared to the plasmid only control (Ecl8Δ*ramR*/pACYC177) (Fig. 2D). In contrast, the levels of sRamA5 were found upregulated by ∼ 2.8 fold in Ecl8Δ*ramR*/pAC*ramR* relative to the plasmid only control (Ecl8Δ*ramR*/pACYC177). Thus the increase in sRamA5 levels in the presence of a functional *ramR* supports our hypothesis that sRamA5 is stabilized by RamR. Our data also shows that sRamA5 does compete with pI for RamR binding, although this effect may be abrogated by the higher relative affinity of RamR to the pI promoter (Fig. 2B(ii)). Therefore, we surmise that the physiological relevance of RamR-sRamA5 interaction supports the basal level of *ramA* transcription detected in the wild type *K. pneumoniae* Ecl8.

### Describing the Transcriptional Landscape of *K. pneumoniae* Ecl8 and Its Isogenic Derivatives Ecl8Δ*ramA*, Ecl8Δ*ramR*

To determine the effect of altered RamA levels on the whole transcriptome of *K. pneumoniae* strain Ecl8, we quantitatively compared the transcriptomes of the three strains (Ecl8, Ecl8Δ*ramA*, Ecl8Δ*ramR*) using the Kolmogorov-Smirnov (K-S) 2-sample test (S3 Fig.) as described in the supplementary data [25]. As expected, the distribution curve of Ecl8 and Ecl8Δ*ramA* were more similar to each other compared to that observed for Ecl8Δ*ramR*, suggesting that under normal growth conditions the deletion of *ramA* is less likely to perturb the transcriptional landscape as opposed to when it is overexpressed. This supports the notion that *ramA* functions as a pleiotropic regulator of gene expression in *K. pneumoniae*.

In all three strains, the 16S and 23S rRNA genes showed the highest number of mapped reads consistent with the lack of depletion for ribosomal RNA. However, pairwise comparisons of the normalized basemean values associated with these ribosomal regions were not differentially expressed between Ecl8 and Ecl8Δ*ramR* or Ecl8 and Ecl8Δ*ramA*. The lack of differential ribosomal gene expression is contrary to previous observations in *Salmonella enterica* serovar Typhimurium [4]. Other non-ribosomal genes (e.g. *fusA*_1 (encoding translation elongation factor G), *atpA* (producing ATP synthase F1, α subunit) and *aceE* (encoding a pyruvate dehydrogenase)) were also found to have significantly high basemean values relative to most other genes within the genome. The increased expression of these genes is perhaps not surprising as *atpA* is associated with aerobic growth and *aceE* catalyses the production of precursors to the TCA cycle.

Potential regions of antisense transcription were also detected. However, in most cases, these regions appeared as antisense because of *in silico* errors in annotation or due to transcriptional noise from flanking genes within the chromosome. We did, however, identify antisense transcription, such as with BN373_16241 (producing an oxidoreductase) and BN373_02611, which were differentially expressed due to either elevated RamA levels or loss of the *ramA* gene (S4 Fig.). Coverage plots analyses indicate that the transcription associated with BN373_02611 may be associated with 3’UTR runoff transcription from the divergently transcribed *treBC* operon, in contrast to BN373_16241, which is upregulated when *ramA* was overexpressed and may be a “true” antisense RNA (S4 Fig.).

Genome analyses of *K. pneumoniae* strain Ecl8 [23] identified 11 unique predicted prophage genes encoding phage structural components (BN373_03311, BN373_09871, BN373_10091, BN373_14801, BN373_14811, BN373_14821, BN373_14841, BN373_14921, BN373_21511, BN373_37361, BN373_37371) which were not found to be differentially transcribed in the pairwise comparisons tested (Ecl8 vs Ecl8∆*ramA*, Ecl8 vs Ecl8∆*ramR* (S1 Table). However, pairwise comparisons of Ecl8Δ*ramA* and Ecl8Δ*ramR* detected the differential expression of Ecl8-genome specific genes, BN373_33401, BN373_33411, which were repressed (∼2–3 fold) in the *ramA* overexpressing strain Ecl8Δ*ramR* (S2 Table). Of note, no differential gene expression was noted in the 233 plasmid-coding genes in the *ramA* null mutant or in the *ramA* overexpressor (Ecl8Δ*ramR*) with respect to the wild type (Ecl8).

### Defining the RamA Regulon

**Figure 3:**
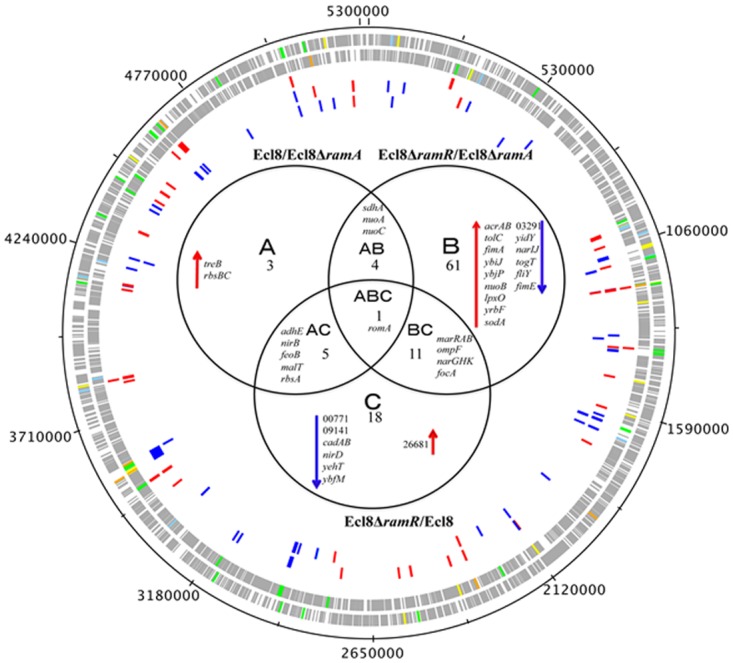
Venn diagram representing the RNA sequencing results. Ecl8Δ*ramA* or Ecl8Δ*ramR* were used as calibrators in the pairwise comparisons. The arrows ⬇ indicates a lower than 0.5 fold decrease in transcription compared to calibrator; ⬆ indicates a higher than 2 fold transcription compared to calibrator. The numbers beneath A, B and C indicate the number of transcripts showing higher or lower transcription (based on statistical cut-off) compared to calibrator. The genes under the different categories A, B and C represent pairwise comparisons between Ecl8/Ecl8Δ*ramA*, Ecl8Δ*ramR*/Ecl8Δ*ramA* and Ecl8/Ecl8Δ*ramR* comparison respectively; the genes in Area AB were found to be differentially transcribed in both the Ecl8/Ecl8Δ*ramA* and the Ecl8Δ*ramR*/Ecl8Δ*ramA* comparisons; the genes in Area AC were found to be differentially transcribed in both the Ecl8/Ecl8Δ*ramA* and the Ecl8/Ecl8Δ*ramR* comparisons; the genes found in the area BC were found to be differentially transcribed in both the Ecl8/Ecl8Δ*ramR* and Ecl8Δ*ramR*/Ecl8Δ*ramA* comparisons. The *romA* gene in Area ABC was found to be differentially transcribed in all the three comparisons.

Transcriptome analyses underscores that perturbations in RamA levels can result in the differential expression of open reading frames, antisense transcripts and Ecl8-specific genes. As the majority of reads were mapped to open reading frames, the main focus of our analyses relates to the differential regulation of genes within *K. pneumoniae*. The RamA regulon in *K. pneumoniae* was identified by pairwise comparisons of Ecl8∆*ramR* versus Ecl8 (C) or Ecl8∆*ramA* (B). The pairwise comparisons of Ecl8 versus Ecl8∆*ramA* (A)(Fig. 3) indicate the cohort of genes (13) responsive to basal levels of RamA expression; the contrast between Ecl8 versus Ecl8∆*ramR* (35) specifies genes that are either affected by RamR or RamA, whereas the comparison between Ecl8∆*ramR* versus Ecl8∆*ramA* (77) identifies genes that largely react to altered RamA levels. As fewer genes are affected due to perturbations in *ramR* expression as opposed to RamA levels, we surmise that the majority of genes differentially expressed in our pairwise comparison (B) are associated with RamA-mediated regulation. Based on this assessment, the probable RamA regulon, Fig. 3, constitutes a total of 103 genes (as in genes in categories A, B, AB, BC, CA, ABC) (S2 Table). Of these, 68 genes were found to be activated and 35 were repressed (S2 Table) when levels of RamA is relatively higher.

Genes associated with RamA-mediated regulation were initially mapped to the COG (clusters of orthologous groups) database to explore their biological function. COG functional classifications of the significantly differentially expressed genes reveal that RamA controls a myriad of cellular and metabolic processes (COG data presented in S2 Table). Generally, altered levels of RamA significantly modulate the expression of genes belonging to the COG functional group C (energy production and conversion). Specifically, when *ramA* is deleted, genes within the COG (G) (carbohydrate metabolism and transport) were also found to be differentially regulated. Pairwise comparison between Ecl8Δ*ramR* versus Ecl8 indicates that COG families associated with transcription (K) and inorganic ion transport and metabolism (P) were also affected. Additionally, when *ramA* levels are elevated genes associated with cell wall membrane and envelope biogenesis (M), transcription (K) and Function UnknowN (FUN) categories were most differentially affected. Thus the resulting COG analyses also supports the observation where altered levels of RamA triggers a shift in gene functionality consistent with significant modulations in transcription patterns as predicted by the K-S test (S3 Fig.).

A closer analyses of the genes associated with pairwise comparisons of Ecl8Δ*ramA* versus Ecl8Δ*ramR* reveals that firstly, the highest number of genes (77) are differentially expressed and secondly genes (*yhbW*, *nfnB*, *acrAB*, *ybhT*, *yrbB-F*) associated with the previously characterized networks for MarA [1], SoxS [26] or Rob [3] in *E. coli* or RamA in *Salmonella enterica* serovar Typhimurium [4, 11] are also affected. This is consistent with previous observations that demonstrate that these proteins exhibit considerable gene overlap within the regulons [1, 4, 11]. Importantly, RamA overexpression results in the modulation of efflux pump genes such as *acrAB*, *oqxAB* and *yrbB-F*, which is consistent with phenotypes linked to multidrug resistance [27] and susceptibility to toxic small molecules, which is associated with alterations in the lipid symmetry of the cell wall [28]. However, the pairwise comparisons for Ecl8 and Ecl8Δ*ramA* also suggest that basal levels of RamA are sufficient to trigger the upregulation of genes such as the trehalose transporter operon *treBC* and the ribose ABC transporter, *rbsACB*. Uniquely, genes associated with biofilm formation (*hha-ybaJ* encodes a toxin-antitoxin system) and lipid A biosynthesis BN373_10601 (encodes lipid A biosynthesis lauroyl acyltransferase, *lpxL_2*) and the related dioxygenase protein encoding gene *lpxO* (BN373_36331) were also found to be upregulated by RamA.

A total of 51 genes were found to be downregulated. As expected, *ompF* was significantly repressed in the *ramA* overexpresser (Ecl8∆*ramR*) (Fig. 3) in addition to genes encoding the nitrate reductases (*narGHJI* operon and *nirD*), BN373_05601 encoding the LysR-type transcriptional regulator, elongation factor EF2 and the riboflavin synthase encoding gene *ribH* were also found to be significantly downregulated in the *ramA* overexpresser (Ecl8∆*ramR*).

**Figure 4:**
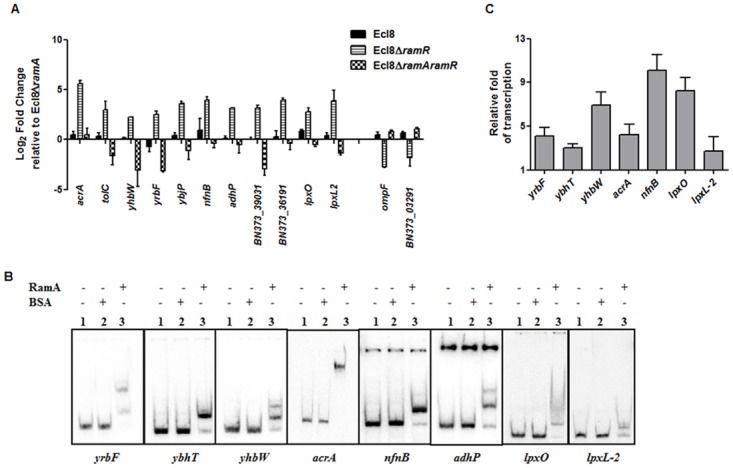
A: Quantitative real-time RT-PCR validation of differentially expressed genes in Ecl8∆*ramR*. All qPCR experiments were performed as outlined in materials and methods. Expression levels were normalized to 16S levels, and fold change values were generated by calibrating against Ecl8∆*ramA*. Genes designated BN373_36191, BN373_39031, BN373_03291 encode a putative membrane protein, oxidoreductase family and conserved hypothetical protein respectively. All data is a mean of 3 experiments. **B:** Electrophoretic Mobility Shift Assay (EMSA) using purified RamA protein. Following PCR amplification, each promoter region was end-labelled with ^32^P-γ ATP. Purified RamA (200 nM) and the different labelled DNA probes (2 nM) were incubated on ice. All reactions were performed on ice prior to electrophoresis on 7.5% native gel. Lane 1 of each panel indicates the labelled DNA probe only, Lane 2 is the BSA control and Lane 3 contains RamA+DNA. **C:** Transcription *in vitro* assay of different promoters using the purified RamA protein. The test DNA (2 nM- *yrbF*, *ybhT*, *yhbW*, *acrA*, *nfnB*, *lpxO* and *lpxC*) with the control template (*gnd*) were incubated for transcription *in vitro* [^32^P]α-UTP with (+) or without (-) 200 nM purified RamA. Samples were fractionated by polyacrylamide/urea gel electrophoresis prior to drying and exposure to the phosphorimager. Relative fold increase was determined using densitometric analysis as described previously [55], by first normalizing all test transcription levels to the control promoter (*gnd*) prior to comparison to the no protein control. Statistics was done using One way ANOVA (P value < 0.05) where transcription levels were found to be statistically significant in the presence of purified RamA compared to the no protein control.

Only a subset of those differentially regulated genes was chosen for validation using qPCR. As expected, both the *romA* and *ramA* genes were found to show 5.25-log_2_ fold and 14.5- log_2_ fold increase in Ecl8∆*ramR* respectively compared to Ecl8∆*ramA* (S5A Fig.). When the activated genes (with the exception of *romA*, *ramA*) were assessed, increased expression of the following genes was noted (Fig. 4A): *tolC* (4.8- log_2_ fold), *acrA* (4.6- log_2_ fold), *yhbW* (1.8- log_2_ fold), *yrbC* (2.8- log_2_ fold), *nfnB* (3.3- log_2_ fold), *ybjP* (3.95- log_2_ fold), *adhP* (3.2- log_2_ fold), BN373_36191 (encodes putative membrane protein, 2.95- log_2_ fold), BN373_39031 (encodes oxidoreductase, aldo/keto reductase family, 1.5- log_2_ fold), *lpxO* (2.8- log_2_ fold) and *lpxL-2* (3.6- log_2_ fold). As expected, the levels of the *ompF* (4.2- log_2_ fold down) and BN373_03291 (encodes conserved hypothetical protein, 1.1- log_2_ fold down) were also downregulated (Fig. 4A).

### Direct Regulation by RamA

In order to determine if some of these differentially expressed genes were under the direct or indirect control of RamA, we performed both EMSA and *in vitro* transcription (IVT) using purified recombinant RamA protein. The EMSA results show that RamA directly binds the *yrbF*, *ybhT*, *yhbW*, *acrA*, *nfnB*, *adhP*, *lpxO* and *lpxL-2* promoters (Fig. 4B). Of note, our controls, showed no shift in the presence of the test promoters (Fig. 4B).

We then determined whether RamA would directly regulate the different promoters identified. By performing IVT experiments, we initially tested the effects of the RamA protein against the *acrAB* promoter to ascertain if RamA would function correctly as a transcriptional activator. As expected, the purified recombinant RamA activated the *acrAB* promoter directly (Fig. 4C) thereby confirming the biological activity of the purified RamA protein. Subsequently, we assessed the test promoters identified by the EMSA in our IVT assays. The results show that RamA upregulates *yrbF* (4-fold), *ybhT* (3-fold), *yhbW* (6.9-fold), *acrA* (4-fold), *nfnB* (10-fold), *lpxO* (8-fold) and *lpxL-2* (∼3-fold) (Fig. 4C). Thus purified recombinant RamA alone can directly activate the expression of these promoters *in vitro*.

### Functional Relevance of RamA-Mediated Overexpression

**Figure 5:**
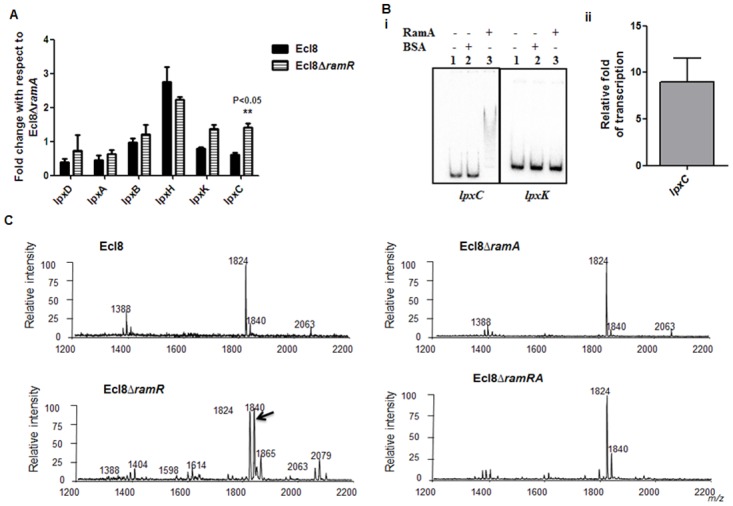
A. Gene Expression analyses of *lpx* genes. All qPCR experiments were performed as outlined in materials and methods. Expression levels were normalized to 16S levels, and fold change values were generated by calibrating against Ecl8∆*ramA*. Two-way ANOVA analyses (P<0.05) were performed to demonstrate statistical significance. **B.** Regulation of *lpx* genes. (i) EMSA using *lpxC* and *lpxK* promoter regions. Purified RamA (200nM) and the different labelled DNA probes (2 nM) were incubated on ice. All reactions were performed on ice prior to electrophoresis on 7.5% native gel. (ii) Transcription *in vitro of lpxC* promoter region. Relative fold increase was determined using densitometric analysis as described previously [55]. Fold increases in the presence of RamA were determined by first normalising to the control promoter (*gnd*) prior to comparison to the no protein control. **C:** Lipid A analysis from *K. pneumoniae* Ecl8 (WT), Ecl8Δ*ramA*, Ecl8Δ*ramR* and Ecl8Δ*ramRA*. Lipid A analysis was undertaken as described before [31]. Negative ion MALDI-TOF mass spectrometry of lipid A isolated from *K. pneumoniae* Ecl8 and its derivatives. Peaks in bold correspond to LpxO dependent 2’ secondary chain modifications.

**RamA regulates genes involved in lipid A biosynthesis**. Having established that purified RamA directly binds and activates the expression of *lpxL-2* and *lpxO* gene promoters (Fig. 4B and 4C), we sought to determine whether RamA could regulate other genes associated with the lipid A biosynthetic pathway. The lipid A biosynthetic pathway is governed by nine enzymes encoded by *lpxA*, *lpxC*, *lpxD*, *lpxB*, *lpxK*, *lpxl*, *lpxM and lpxO* genes [29]. Gene expression analyses using qPCR showed that with the exception of *lpxC*, none of the other *lpx* genes showed significant differential expression in Ecl8Δ*ramR* in comparison to Ecl8 or Ecl8Δ*ramA* (Fig. 5A). We then chose to assess whether RamA would directly interact with the *lpxC* and *lpxK* promoter regions. Subsequent EMSA analyses demonstrate that RamA directly interacts with the *lpxC* but not the *lpxK* promoter (Fig. 5Bi) and increased *lpxC* transcription (9-fold) in the presence of purified RamA and RNA polymerase (Fig. 5Bii). Previous work has shown that the control of lipid A biosynthetic genes is mediated by the PhoPQ or PmrAB systems [30]. Further interrogation of the transcriptome data and subsequent qPCR analyses shows that the levels for *phoP* and *pmrA* levels remained unchanged in *K. pnuemoniae* Ecl8, Ecl8Δ*ramA* and Ecl8Δ*ramR*. Thus the differential modulation of the *lpxO*, *lpxC* and *lpxL-2* genes is directly linked to increased RamA levels.

To ascertain whether RamA-mediated transcriptional activation of *lpxC*, *lpxL*-2 and *lpxO* would actually result in modifications within the lipid A moiety, we performed MALDI TOF mass spectrometry (S1 Text for details). The mass spectrometry analyses confirm alterations in lipid A structure of the *ramA* overexpresser, Ecl8Δ*ramR* compared to the wild type (Ecl8), the null mutant (Ecl8Δ*ramA*) or the double mutant (Ecl8Δ*ramRA*) (Fig. 5C) where peaks (m/z 1840, 1866 and 2079) were found to be elevated. Previous studies in *K. pneumoniae* [31, 32] indicate that those peaks correspond to LpxO hydroxylated lipid A species containing a hydroxymyristate group at position 2’ as secondary acyl substitution. Therefore, we surmise that RamA mediated activation of the different lipid A biosynthetic genes leads to alterations within the lipid A moiety in *K. pneumoniae*.

### Antibiotic Susceptibility

**Phenotype microarray analyses**. In order to assign phenotypes linked to the differentially regulated genes, Biolog phenotype assays were undertaken for *K. pneumoniae* Ecl8 and its isogenic derivatives Ecl8Δ*ramA* and Ecl8Δ*ramR*. A comparison of Biolog phenotypic profiles of both *Salmonella* [11] and *K. pneumoniae* generally indicates a significant overlap in the susceptibilities to antimicrobial and toxic compounds (S3 Table). As expected, the overexpression of *ramA* resulted in increased tolerance of Ecl8Δ*ramR* in the presence of antimicrobials such as tetracyclines (doxycycline, chlortetracycline, minocycline), macrolides (erythromycin, spiramycin, troleandomycin), beta-lactams (1st, 2nd, 3rd generation cephalosporins, penams) and (fluoro)quinolones (ciprofloxacin, ofloxacin, nalidixic acid, novobiocin), fungicides (such as chloroxylenol, dodine, domiphen bromide) and toxic anions (potassium tellurite, sodium metasilicate) (S3 Table, S6 Fig.). Notably, comparisons of the Biolog data also indicate that *ramA* overexpression results in altered polymyxin B susceptibility levels in both *K. pneumoniae* and *Salmonella*.

### Susceptibilities to the Polymyxins and the Cationic Antimicrobial Peptides (cAMPs)

**Figure 6:**
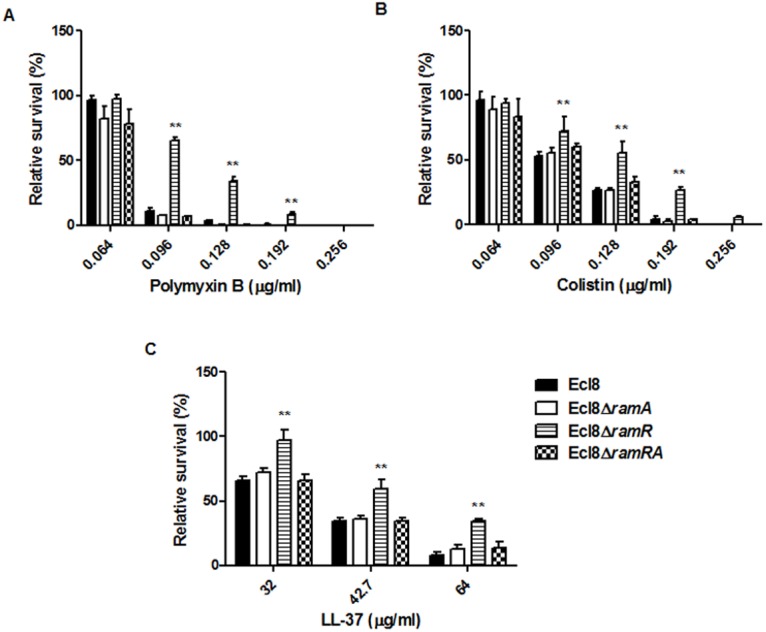
Survival assay of *K. pneumoniae* (Ecl8, Ecl8∆*ramA*, Ecl8∆*ramR*, Ecl8∆*ramRA*) to polymyxin B, colistin and the antimicrobial peptide LL-37. The relative survival of bacteria (expressed as a percentage of the number of colonies obtained from the unexposed control of the same strain) in the presence of different concentrations of polymyxin B (A), colistin (B) and LL-37 (C) are shown. Asterisks indicate that results obtained for the *ramA* expresser, Ecl8Δ*ramR* is significantly different (P < 0.05 by Two-way ANOVA) compared to Ecl8, Ecl8Δ*ramA* and Ecl8∆*ramRA*.

Lipid A synthesis in Gram-negative bacteria is controlled at both the transcriptional and translational levels, where alterations in the lipid A profile can result in perturbations in host-microbe interactions as well as reductions in susceptibility to both the polymyxins and the cationic antimicrobial peptides (cAMPs) [33]. Accordingly, we tested the strain Ecl8 and its isogenic derivatives Ecl8Δ*ramA*, Ecl8Δ*ramR* against colistin, polymyxin B and the cAMP LL-37. The relative survival assays for colistin, polymyxin B and LL-37 demonstrated that the *ramA* overexpressing strain, Ecl8Δ*ramR* strain was significantly (P < 0.05) less susceptible to polymyxin B, colistin and LL-37 (Fig. 6 A, B, C) compared to the wild type Ecl8 and the null mutant Ecl8Δ*ramA*. The reduction in polymyxin susceptibility, as noted in the survival assays, is also supported by the Biolog data (S3 Table). Taken together these results suggest that RamA-dependent regulation provides an alternative pathway for reduced susceptibility to polymyxins and cAMPs.

### Effect of RamA Overexpression on Host-Microbe Interactions

**Figure 7:**
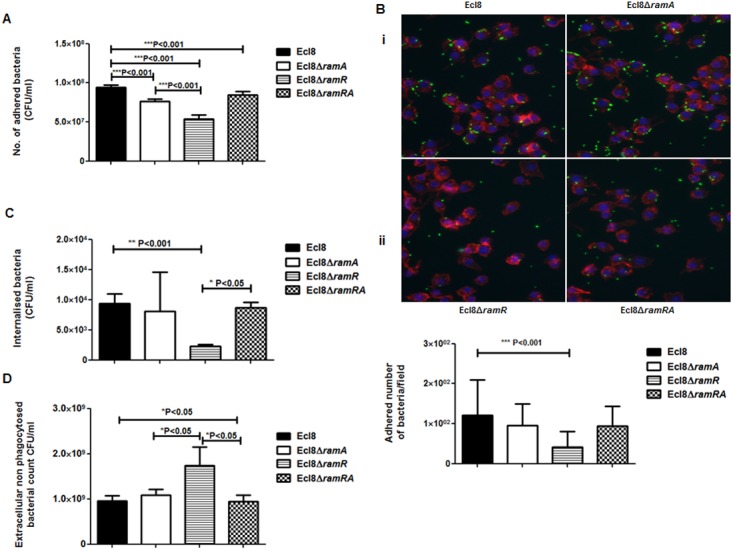
A: Attachment of *K. pneumoniae* Ecl8, Ecl8Δ*ramA*, Ecl8Δ*ramR* or Ecl8∆ramRA to murine macrophage RAW 264.7 cell line. One-way ANOVA analyses were performed to demonstrate statistical significance. **B:** Microscopy to assess attachment to RAW 264.7 cell line. (i) Infection of the RAW264.7 cell line was carried out with *K. pneumoniae* Ecl8 (WT), Ecl8Δ*ramA*, Ecl8∆*ramR* or Ecl8Δ*ramRA* transformed with plasmid pRSMgfp. MOI was 1:100 and infections were carried out for 2 hrs. The actin cytoskeleton was stained with Acti stain 555 phalloidin (red) and host cell nuclei were stained with DAPI (blue). Images are representative of 80 fields. (ii) Graph representating mean values are derived from 3 independent experiments. One-way ANOVA analyses (P<0.001) were performed to demonstrate statistical significance. **C:** Internalisation of *K. pneumoniae* Ecl8, Ecl8Δ*ramA*, Ecl8Δ*ramR* or Ecl8∆*ramRA* by RAW 264.7 cells. Bacterial internalisation was assessed by the gentamicin protection assay. One-way ANOVA analyses were performed to demonstrate statistical significance. **D:** Enumeration of the extracellular non-phagocytosed *K. pneumoniae* Ecl8, Ecl8Δ*ramA*, Ecl8Δ*ramR* or Ecl8∆*ramRA*. One-way ANOVA analyses were performed to demonstrate statistical significance.

**Macrophage-*Klebsiella* interaction**. To ascertain whether RamA-mediated alterations can have an impact on microbe-macrophage interactions, we examined if Ecl8 and its isogenic derivatives, Ecl8Δ*ramR*, Ecl8Δ*ramA* and Ecl8Δ*ramRA* would exhibit differential interactions in adherence and intracellularization into murine RAW macrophages. In the adhesion and intracellularization assays, the *ramA* overexpresser, Ecl8Δ*ramR*, was significantly attenuated in its ability (approximately 50% decrease) to attach to and internalise into the RAW murine macrophage cells compared to wild type *K. pneumoniae* Ecl8, the mutants Ecl8Δ*ramA* and Ecl8Δ*ramRA* (Figs. 7A, B and C). Two possible explanations exist for the reduction in adherence and intracellularization of Ecl8Δ*ramR*; the first, where altered RamA levels confers resistance to phagocytosis and the second, is due to accelerated killing by the macrophage. In order to ascertain whether the reduced intracellularization of Ecl8Δ*ramR* was linked to accelerated killing by macrophages, we determined the levels of extracellular non-phagocytosed bacteria in our experiments and found significantly higher numbers of recovered bacteria for Ecl8Δ*ramR* compared to the wild type Ecl8, Ecl8Δ*ramA* and Ecl8Δ*ramRA* (Fig. 7D). In previous work [34], resistance to phagocytosis by *K. pneumoniae* has been linked to bacterial surface structures which include the capsular polysaccharide (cps). However, *ugd* gene transcription, representative of the *cps* cluster [35], was not found to be altered in Ecl8, Ecl8Δ*ramA*, Ecl8Δ*ramR* and Ecl8Δ*ramRA* (S5B Fig.), consistent with the RNAseq data. Thus our results underscore that reduced phagocyte adhesion and uptake is linked to RamA-mediated alterations, particularly those associated with lipid A.

### Infection *In Vivo*

**Figure 8:**
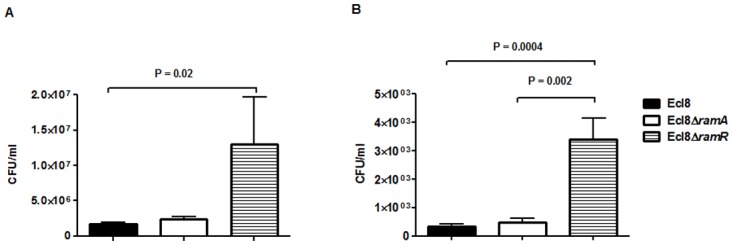
Effect of RamA on bacterial recovery using an intranasal infection model. Bacterial recovery (cfu/ml) was determined from lung (A) and spleen (B) homogenates following a 24h infection of 5–7 week old, C57BL6, female mice (n = 5) using the previously described intranasal infection model. Unpaired t-test analyses were performed to demonstrate statistical significance.

In order to assign a broader relevance to altered *Klebsiella*-host interaction, we performed experiments to assess bacterial recovery using the intranasal inoculation method [36] as described previously. Following a 24-hour infection of 5–7 week old C57BL mice, organ homogenates (spleen and lung) were plated to determine bacterial counts. At 24 h post infection, bacterial recovery rates for the *ramA* overexpressor, Ecl8Δ*ramR* were found to be significantly higher compared to the wild type Ecl8 or null mutant Ecl8Δ*ramA* from the lung and spleen (Fig. 8(A) and 8(B)). The intranasal route of infection is expected to result in the primary infection of the lung prior to dissemination to other organs. Our results demonstrate that significantly higher levels of Ecl8Δ*ramR* is recovered from both the lung and spleen highlighting that RamR-dependent RamA overexpression, confers reduced microbial clearance and increased systemic dissemination of *K. pneumoniae* in an intranasal infection model.

## Discussion

The relevance of the MarA, SoxS, Rob, RamA and RarA regulators in microbial survival is attributed to their control of the antimicrobial resistance phenotype in a wide variety of Gram-negative bacteria [10, 37, 38]. Whilst the role of RamA in reduced antibiotic susceptibility is evident from multiple studies [16, 17, 37], its broader role in gene regulation is not known in *Klebsiella pneumoniae*. Using transcriptome profiling, we demonstrate that RamA-overexpression results in altered *K. pneumoniae* transcription patterns (S3 Fig.) compared to the null mutant or wild type strain thus highlighting its wider role in gene regulation in *K. pneumoniae*.

Our data suggests that RamA functions largely as a transcriptional activator of gene expression, where DNA-binding (Fig. 4B) and IVT assays (Fig. 4C) demonstrate that this regulation is direct and likely mediated via a *mar*/*ram*-box like element [39] located within the promoter region. Whilst our work is the first to demonstrate direct RamA-mediated activation of gene expression, other studies have shown that related proteins such as MarA, SoxS [40] and RarA [5] also exert explicit control of regulon genes. Comparative transcriptome data analyses suggests that RamA-mediated activation is dependent on regulator concentration (basal versus overexpressed, Fig. 2) in addition to the observation that identical RamA levels induce differential levels of promoter activation as supported by our *in vitro* data (Fig. 4C). The maintenance of basal *ramA* levels may be necessary for the *K. pneumoniae* stress response to a variety of agents as has been previously shown when selecting for fluoroquinolone resistant *Salmonella* [41] *or Klebsiella* in a *ramA*-deleted strain. In *K. pneumoniae*, basal levels of *ramA* expression is maintained due to titration of the absolute repressory effects of RamR by the RamR-sRamA5 interaction (S2 Fig.). Uniquely for tetracycline family regulators, RamR, directly interacts with the regulatory RNA, sRamA5, (Fig. 2B) which is produced as a cleaved by-product of the primary *romA* transcript (Fig. 2B). Whilst the sRamA5-RamR interaction, provides basal levels of *ramA* expression, *ramA* transcription as observed in the overexpressor, Ecl8Δ*ramR* or clinical strains [16] are linked to loss of function mutations within RamR. Consequently, our data show that the maximal changes in gene expression profiles are observed when *ramA* is overexpressed as in Ecl8Δ*ramR* (S3 Fig.). In this gene cohort, we demonstrate that RamA impacts on gene transcription linked to operons associated with efflux pumps, biofilm formation and lipid A biosynthesis (Fig. 3, S2 Table). Whilst it is possible that the differential regulation of these genes is not all directly linked to RamA, we demonstrate that purified RamA directly binds and activates the expression of multiple associated promoters (Fig. 4C & 4D).

A comparison of RamA-mediated regulation in *Salmonella enterica* serovar Typhimurium [4] and *K. pneumoniae* establishes key similarities in the genes associated with the respective RamA regulons; particularly in the control of genes associated with antimicrobial resistance *acrAB* and *ompF* [4, 11]. Additionally, RamA-dependent direct activation of *acrAB* is also consistent with phenotypic studies [10, 16–18] which consistently demonstrate that *ramA* overexpression is linked to increased elevated efflux via *acrAB* and decreased outer membrane protein levels (OmpF). Given its role in conferring reduced antimicrobial susceptibility, it is perhaps not surprising that we demonstrate that RamA directly regulates other efflux related operons specifically; the AcrAB linked inner periplasmic protein, YbhT [42] associated with detergent sensitivity, the Yrb operon which encodes an ABC transporter linked to the export of quinolones [27] and also lipid asymmetry [30]. The combined effect of the efflux or influx levels and membrane alterations associated with transport and structural variations likely contributes to the substrate range of compounds impacted by *ramA* overexpression (S3 Table). However, in the absence of a functional *acrAB* efflux pump, RamA-overexpression does not confer reduced susceptibility to most antibiotics in *K. pneumoniae*. This observation is consistent with previous studies for the MarA and RarA [38] proteins.

Therefore, it is likely that a functional AcrAB pump is crucial in mediating decreased antimicrobial susceptibility. However, a recent study [43] also suggests that *acrAB* may play a role in decreased antimicrobial peptide susceptibility and increased virulence in *K. pneumoniae*. Our findings support this observation and further demonstrate that increased RamA levels can also mediate LPS alterations, which likely contribute towards increased survival to both polymyxins and cationic AMPs (Fig. 5, 6).

Structurally, LPS is composed of three domains, the serovar dependent O-antigen chain, core oligosaccharide consisting of sugars and lipid A which is a phosphorylated disaccharide decorated with multiple fatty acids which anchor the LPS into the bacterial membrane [29]. The endotoxic lipid A component of LPS constitutes the outermost layer of the outer membrane of Gram-negative bacteria thereby playing a critical role in host-microbe interactions in addition to promoting reduced susceptibility to cAMPs [44] such as polymyxins [30] and host derived factors LL-37, HBD-1 [30]. Studies have shown that lipid A modifications can result in multiple outcomes such as reduced polymyxin susceptibility [45] in addition to directly facilitating microbial evasion by reduced immune recognition [46]. Our work suggests that the molecular basis for the modified lipid A structure is linked to the differential regulation of the biosynthesis genes e.g. *lpxO*, *lpxL*-2 and *lpxC* identified in this screen. Despite being constitutively produced the regulation of *lpxC*, *lpxL*-2 and *lpxO*, is still subject to either transcriptional or translational control [44, 46]; generally in response to stress, where, *lpxC* and *lpxL*-2 are regulated by the two-component systems, PhoPQ and PmrAB [44]. In contrast, *lpxO* is not subject to PhoPQ regulation in *Salmonella* [44, 46].

In *Salmonella* Typhimurium, the modulation of LpxO levels results in the remodeling of the outer membrane which reduces the net negative charge whilst simultaneously increasing membrane integrity resulting in increased virulence [47]. A similar phenotype is exhibited by the *K. pneumoniae* Ecl8Δ*ramR* strain, which has altered LpxO levels (Fig. 8). Thus we surmise that the altered host-microbe and polymyxin-microbe interactions are in part attributable to the lipid A modifications.

Macrophages represent a key innate host defence strategy against microbial infections as phagocytosis of incoming pathogens is a trigger for the inflammatory response. Our data show that *ramA* overexpression protects against macrophage uptake and internalization (Fig. 7) thus providing a basis for the greater dissemination of the *ramA* overexpressing strain, Ecl8Δ*ramR* in an *in vivo* infection model. Taken together, these RamA-linked phenotypes underscore its’ role in *Klebsiella* virulence and survival *in vivo*.

The molecular basis for phenotypes associated with reduced antimicrobial peptide susceptibility and increased virulence can be attributed to several key loci such as the *acrAB* pump and lipid A biosynthesis genes, *lpxC*, *lpxL*-2 and *lpxO*. This is supported by studies that demonstrate the involvement of *acrAB* [48] and lipid A modifications [30, 44] in host-microbe interactions. However to definitively pinpoint the exact contribution of the lipid A biosynthetic genes or *acrAB* to phenotypes associated with host-pathogen interactions would require the deletion of genes encoding *lpxC* [49], *lpxL*-2 and *lpxO* [50], *acrAB* individually or in combination with *ramA* overexpression. We note that previous studies [32, 50] have shown that strains deleted for these genes, result in avirulent microbes and as such, this phenotype would obscure any RamA-associated effects. Nevertheless, our work is first to demonstrate that firstly, RamA functions as an alternate regulator of certain lipid A biosynthesis genes and secondly, these alterations perturb microbe-host interaction.

The significance of our findings lies in the broader implications of RamA-mediated regulation in Enterobacteriaceae. In this work, we describe roles for RamA in both protection against antibiotic challenge but also against the innate host immune response thus resulting in *Klebsiellae* that are less susceptible to antibiotics and simultaneously more virulent. Notably, our findings highlight that RamA mediated overexpression via both increased *acrAB* expression and lipid A alterations can result in reduced susceptibility to the last line drugs e.g. tigecycline and polymyxins. This highlights the broader consequences in selecting for *ramA* overexpression in *K. pneumoniae* or other members of *Enterobacteriaceae*. Finally, our study underscores and highlights the importance of intrinsic proteins such as RamA, which regulate survival strategies in *K. pneumoniae* and likely other Enterobacteriaceae, specifically in priming the microbial population in surviving drug and host immune pressure. This proposes the notion where microbial immune evasive strategies contribute to the development and persistence of antimicrobial resistance.

## Materials and Methods

### Growth Conditions

Bacteria (Table 1) were cultured in Luria-Bertani (LB) medium (10 g/L tryptone, 5 g/L yeast extract, 10 g/L NaCl). Typically, a strain was first grown on an LB plate at 37°C from frozen -80°C stocks. A single colony was picked and inoculated into 5 ml of LB and incubated in a 37°C shaker overnight. A 1 in 100 dilution was made in LB and incubated in a 37°C shaker until the OD_600_ reached 0.6 unless otherwise stated. Antibiotics such as ampicillin (100 µg/ml) and chloramphenicol (20 µg/ml) were added as required.

**Table 1:** Strains used in this study.

**Strains**	**Description**	**Ref**
Ecl8	Wild type *K. pneumoniae*	[59]
Ecl8Δ*ramA*	*K. pneumoniae* deleted of *ramA*	[16]
Ecl8Δ*ramR*	*K. pneumoniae* deleted of *ramR*	[16]

### Antimicrobial Susceptibility and Peptide Survival Assay

The assay was as described previously by Moranta *et al* [51]. Briefly, bacteria were grown at 37°C in 5 ml LB medium, harvested (5,000 × g, 15 min, 5°C) and washed thrice with phosphate-buffered saline (PBS). A suspension containing approximately 10^5^ CFU/ml was prepared in 10 mM PBS (pH 6.5), 1% tryptone soy broth (TSB; Oxoid), and 100 mM NaCl. Aliquots (5 μl) of this suspension were mixed in tubes with various concentrations of polymyxin B, colistin (0.064 µg/ml to 0.256 µg/ml) and LL-37 (32 µg/ml to 85.3 µg/ml) to a final volume of 30 µl. Following incubation for an hour at 37°C with polymyxin B (Sigma, UK), colistin (Sigma, UK) and LL-37 (Sigma, UK) the samples were diluted 1:10 with PBS prior to plating (100 μl) on LB agar. Colony counts were determined after overnight incubation, where results are expressed as percentages of the colony count of bacteria that were not exposed to the antibiotics or the antimicrobial peptide. Sensitivity profiles of the different mutants using the phenotypic microarray analyses were determined described in S1 Text.

### RNA Extraction, RNA-Seq Sample Preparation and Sequencing

Overnight cultures of strains Ecl8, Ecl8Δ*ramA*, Ecl8Δ*ramR* were inoculated (1/100 dilution) into LB media and incubated at 37 ºC with vigorous shaking. Cell pellets were harvested at OD_600_ = 0.6 and RNA was extracted using the RNAeasy Extraction Kit (Qiagen, Hilden, Germany), which enriches for RNA molecules larger than 200 nucleotides. No depletion of ribosomal RNA was carried out prior to the synthesis of single-stranded cDNA (sscDNA) as previously reported [52]. RNAseq DNA libraries were constructed as previously described [53]. For RNAseq, independent biological samples in triplicate were assessed for each strain. The resulting sscDNA libraries were sequenced in an Illumina HISeq 2000 sequencer. An average of 0.715 Gb of sequence data was obtained per sample, in 75 bp paired reads (Details of RNAseq analyses are outlined in S1 Text). The RNAseq read data has been deposited under the ENA data repository and ArrayExpress with the accession numbers ERP001994 and E-ERAD-122, respectively.

RNA for quantitative Real-Time PCR experiments was extracted from *K. pneumoniae* strains (Table 1) using the TRIzol extraction method [16]. Briefly, cells were grown to mid-log phase (OD_600_ = 0.6) at 37 ºC with shaking and then harvested by centrifugation at 3000g (PK121R, ALC) at 4 ºC. The cell pellet was then resuspended in TRIzol reagent (Invitrogen, Paisley, UK) and chloroform prior to centrifugation to separate the phases. The upper phase was then precipitated using 3 M sodium acetate, glycogen (5 mg/ml), and 100% ethanol.

Both RNA preparations were washed and resuspended in 50 µl DEPC treated water. RNA was treated with TurboDNase to remove DNA contamination (Ambion, New York, USA). All samples were assessed for RNA integrity and quantification using both the Bioanalyzer 2100 RNA nanochip (Agilent, UK) and the ND-1000 (Nanodrop Technologies) [4]. Only those samples with integrity level 9 were taken forward for library construction or qPCR analyses.

### Quantitative Real-Time PCR (qPCR)

In order to validate the RNAseq data, quantitative Real-Time PCR experiments were undertaken. After the removal of contaminating DNA, cDNA synthesis was generated using the AffinityScript cDNA synthesis kit (Agilent, UK). Gene specific primers (S4 Table) were designed using the Primer3 (http://frodo.wi.mit.edu/) software and were tested to produce standard curves with amplification efficiencies ranging from 95–110%. qPCR analyses using the locked nucleic acid probe is detailed in S1 Text. Quantitative Real Time RT-PCR (RT-PCR) was performed using the synthesized cDNA with gene specific primers using the Brilliant III Ultra-fast SYBR Green Kit (Agilent, UK) in the Agilent Mx3005P. All data were analyzed using Agilent MxPro software, which is based on the efficiency corrected method (Pfaffl) of comparative quantification that utilizes the ΔΔCt approach, also taking into account primer efficiency. The relative fold increases in expression levels were determined by firstly normalizing gene expression levels to 16S rDNA and using either Ecl8 or Ecl8∆*ramA* as calibrators. All comparative analyses were done using the MxPro software (Agilent, UK).

### DNA EMSA

DNA fragments that represent the promoter regions of the genes that were differentially regulated in the presence or absence of RamA or RamR were subjected to the electrophoretic gel shift mobility assay (EMSA) as described previously [54]. Briefly, DNA templates ranging from 250–150bp upstream of the start site were produced by PCR, and purified by StrataPrep PCR Purification kits (Agilent UK). The purified templates were end-labelled with [γ^32^P]-ATP by T4 Kinase (New England Biolabs, USA). The unincorporated, labelled ATP was removed using Biospin P6 spin columns (Biorad, UK) as per manufacturer’s instructions. Purified RamA was extracted from the recombinant pET*ramA* construct using metal chelation chromatography on superflow nickel / nitrilotriacetate agarose (Qiagen, Germany) (James Hastie, Dundee University). His-tagged RamA (200 nM) and labelled DNA (2 nM) were mixed in binding buffer (125 mM Tris-HCl, 250 mM KCl, 5 mM DTT 5% glycerol) and incubated on ice for 15 min prior to electrophoresis at 75 V on a prechilled 7.5% native polyacrylamide gel in 1 × TBE buffer.

### Transcription *In Vitro*

Transcription (IVT) experiments were performed as described previously [55]. Briefly 5 × IVT Buffer, 2 nM PCR product of the test and control (*E. coli gnd* [56]) promoters, RNA polymerase, RNAseOUT (Invitrogen, UK) was incubated for 15 minutes at 37°C prior to the addition of the transcription mix containing × 5 IVT buffer (50 mM Tris-HCl, 0.1 mM EDTA, 3 mM magnesium acetate, 0.1 mM dithiothreitol, 20 mM sodium chloride, and 250 μg/ml bovine serum albumin at pH 7.8), heparin (1.2 µg/ml), NTPS, and α^32^P-UTP (Perkin Elmer, UK). The reaction was stopped 5 minutes later followed by the addition of Gel loading buffer II (Ambion, UK). The resulting products were electrophoresed on a 7% polyacrylamide / 8 M urea gel. Quantification was determined by densitometric analysis using Fujifilm Multigauge Software where an increase or decrease in transcription levels is after normalization to the endogenous *gnd* levels and calibration to the no protein control.

### Cell Culture

Murine RAW 264.7 macrophage cells (obtained from ATCC TIB-71) were cultured in Dulbecco’s Modified Eagle Medium (PAA, UK) supplemented with 10% endotoxin-free foetal bovine serum (PAA, UK) and penicillin and streptomycin (Invitrogen, UK) in 75-cm^2^ culture flasks in 5% CO_2_ for 24 h until subconfluent. Twelve well tissue culture plates were seeded with 5 × 10^5^ cells per well and viability determined using trypan blue exclusion.

### Bacterial Adhesion and Internalisation Assays

Bacterial adhesion and internalization experiments were performed as described previously [57, 58]. For the adhesion assays, RAW cells were washed with PBS and incubated for 2 h at 37°C in 5% CO_2_ with a suspension of 5 × 10^7^ bacterial cells in DMEM medium alone. After incubation, wells were washed five times with PBS and adherent bacteria were released by addition of 0.5% Triton X-100 (Sigma, UK). Bacterial colonies were quantified by plating appropriate dilutions on LB agar plates. In the internalization assays, after the incubation of the RAW cells with the bacterial suspension, wells were washed twice with PBS and then incubated for 2 h with fresh DMEM medium containing gentamicin (100 µg/ml) to eliminate extracellular bacteria. After the incubation, an aliquot of the medium was plated to confirm killing of extracellular bacteria and the gentamicin-containing medium was washed again. RAW cells were lysed and intracellular bacteria were quantified as described above. To estimate levels of extracellular bacteria, the infection of the RAW cells was carried out as described previously for the adhesion assay. After incubation, the media with the non-phagocytosed extracellular bacteria was collected and quantified by plating appropriate dilutions on LB agar plates. All microscopy images were generated as outlined in S1 Text.

### Ethics Statement

All mouse experiments were performed under the control of the UK Home Office legislation in accordance with the terms of the Project license (PPL2700) granted for this work under the Animals (Scientific Procedures) Act 1986 in addition to receiving formal approval of the document through Queen’s University Belfast Animal Welfare and Ethical Review Body.

### Infection *In Vivo*

Overnight bacterial cultures were washed three times in sterile endotoxin free PBS. The bacteria was resuspended to an optical density of 0.2 and 20 μl (∼ 5 × 10^7^ CFU/animal) and administered to anaesthetised 5–7 week old weight watched Harlan C57BL6 mice (n = 5 per group) using the intranasal inoculation method [36]. In order to ensure maximal delivery of the bacterial inoculation into the lungs the animals were held in a perpendicular position until cessation of laboured breathing. 24 h post inoculation the mice were sacrificed by lethal pentabarbitol injection. Perfused lungs and spleen were harvested and resuspended in 1 ml of sterile PBS. Following mechanical homogenisation dilutions were plated on LB agar plates and incubated at 37°C to establish the CFU/ml.

## Supporting Information

S1 Fig Transcriptional start sites (TSS) of *ramR*, sRamA5 and *romA* determined by 5’ RACE. Capitalized triplets are start codons of either *ramR* or *romA*. Bold capital letters indicate the primary and secondary TSS sites for *ramR* or *sRamA5* and *romA*. The primary and secondary TSSs of *ramR* are indicated with a black and grey arrow respectively. The shaded segments are the inverted repeat (IR) sequences recognized by the RamR protein. The sequences indicated by the dotted underlined fragment (RL) and the single underlined fragment (RH) were used in eGFP analysis. The numbering system is based on the “t” prior to *romA*’s start codon ATG as the -1.
(TIF)

S2 Fig Fluorescence reporter analysis for RH promoter activity and its repression by RamR. Where indicated DH5α contained both the pKC026 (containing either the RH fragments) and pBR322*ramR* plasmid. DH5α haboring both pKC026 and pBR322 is taken as a negative control and the RH fragment containing pKC026 with pBR322 only taken as the baseline control.
(TIF)

S3 Fig Kolmogorov-Smirnov 2-sample test. The plots show the sample distribution of log_2_-transformed basemean values from the RNAseq experiment for Ecl8, Ecl8Δ*ramA* and Ecl8Δ*ramR*. The D value (the largest vertical distance between two curves) between Ecl8 and Ecl8Δ*ramA* is 0.046; the one between Ecl8 and Ecl8ΔramR is 0.067, and the one between Ecl8Δ*ramA* and Ecl8Δ*ramR* is 0.036. All of the distributions are significantly different from each other (p < 0.001).
(TIF)

S4 Fig Coverage plot for the antisense transcription of BN373_16241 in Ecl8Δ*ramA* and Ecl8Δ*ramR*. The coverage plot is visualized with Artemis Genome Browser using the Ecl8 genome as a reference. Window size is set at 3. One representative coverage plot of Ecl8Δ*ramA* and Ecl8Δ*ramR* is shown here. The borders of the coding region of BN373_16241 are marked by vertical black bars in the coverage plot. The green and red curves within the borders represent the antisense and sense transcription of BN373_16241 respectively.
(TIF)

S5 Fig A: Quantitative real-time RT-PCR analyses of *romA* and *ramA* gene expression levels in Ecl8Δ*ramR*. B: Gene expression of *ugd*, *pagP* genes in different *K. pneumoniae* strains. All qPCR experiments were performed as outlined in materials and methods. Expression levels were normalized to 16S levels, and fold change values were generated by calibrating against Ecl8∆ramA. All data is a mean of 3 experiments.
(TIF)

S6 Fig Heat map of the Biolog phenotype of Ecl8, Ecl8Δ*ramA* and Ecl8Δ*ramR*. Biolog analyses of the wild type *K. pneumoniae* Ecl8, Ecl8Δ*ramA* and *K. pneumoniae* Ecl8Δ*ramR* using PM1–20 plates.
(TIF)

S1 Text Supporting Materials and Methods. (DOCX)

S1 Table Raw mapped RNAseq data. (XLS)

S2 Table List of differentially expressed genes in the pairwise comparison with COG analyses. (XLSX)

S3 Table Biolog phenotypic profile of the *K. pneumoniae* stains. (XLSX)

S4 Table List of primers used in this study. (XLSX)
