# Correction: Elucidation of the RamA Regulon in *Klebsiella pneumoniae* Reveals a Role in LPS Regulation

**DOI:** 10.1371/journal.ppat.1004766

**Published:** 2015-03-19

**Authors:** The PLOS Pathogens Staff

The eighth author’s name is spelled incorrectly. The correct name is: Avril Monahan.
