# Correction: Elucidation of the RamA Regulon in *Klebsiella pneumoniae* Reveals a Role in LPS Regulation

**DOI:** 10.1371/journal.ppat.1005649

**Published:** 2016-05-11

**Authors:** Shyamasree De Majumdar, Jing Yu, Maria Fookes, Sean P. McAteer, Enrique Llobet, Sarah Finn Shaun Spence, Avril Monaghan, Adrien Kissenpfennig, Rebecca J. Ingram, José Bengoechea, David L. Gally, Séamus Fanning, Joseph S. Elborn, Thamarai Schneiders

The authors would like to correct Fig 4 and Fig 7, as errors were made in the preparation of these figures for publication. In Fig 4B, the existing panels for the gene promoters *yrbF* and *ybhT* are the wrong EMSA images for these promoters. The authors have provided a corrected version of Fig 4 here. In Fig 7B, the panel for Ecl8Δ*ram*A was a replication of the image shown for the wild type strain *K*. *pneumoniae* Ecl8. The authors have provided a corrected Fig 7 here.

**Fig 4:**
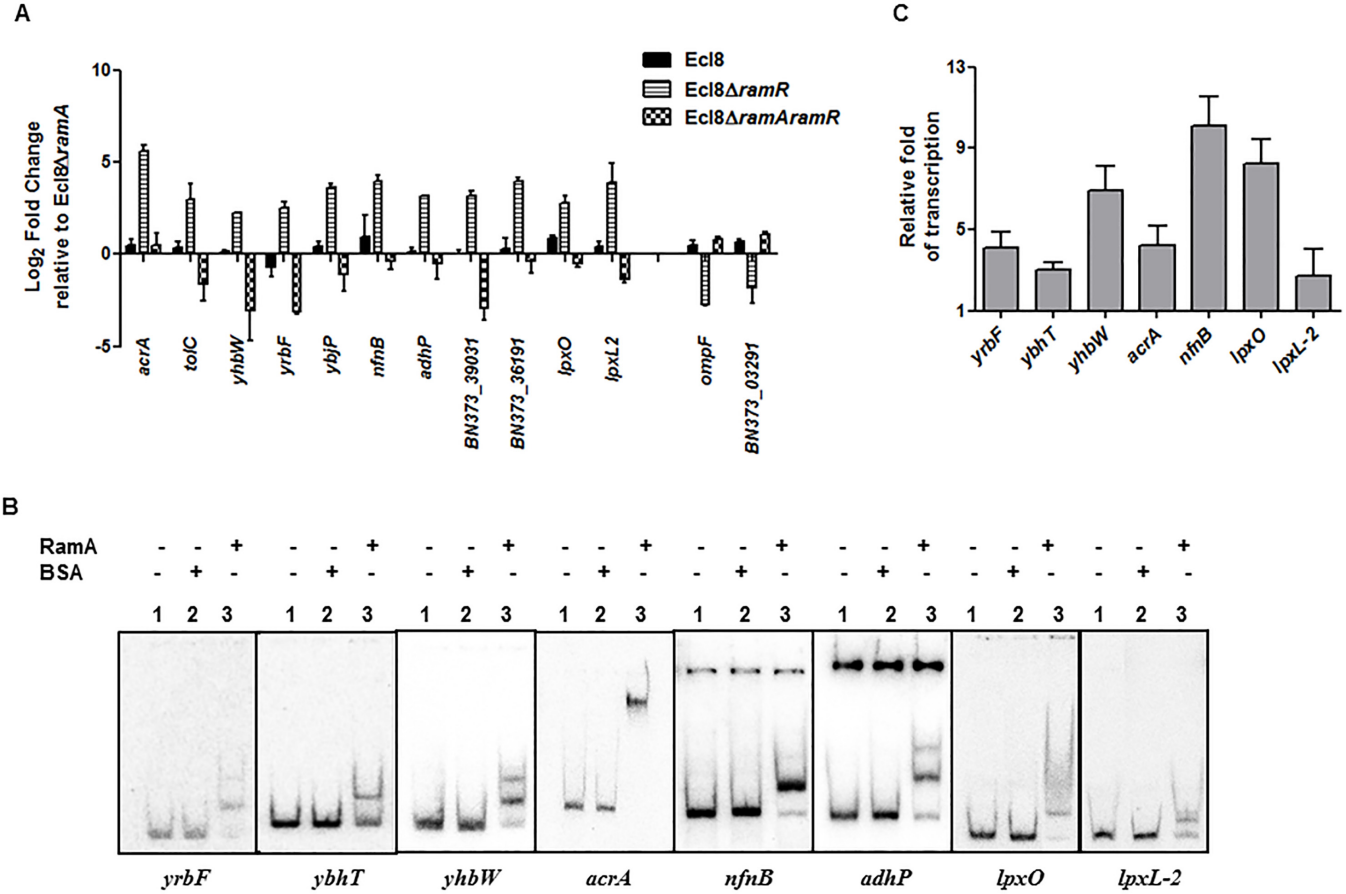
**A: Quantitative real-time RT-PCR validation of differentially expressed genes in Ecl8Δ*ramR*.** All qPCR experiments were performed as outlined in materials and methods. Expression levels were normalized to 16S levels, and fold change values were generated by calibrating against Ecl8Δ*ramA*. Genes designated BN373_36191, BN373_39031, BN373_03291 encode a putative membrane protein, oxidoreductase family and conserved hypothetical protein respectively. All data is a mean of 3 experiments. **B:** Electrophoretic Mobility Shift Assay (EMSA) using purified RamA protein. Following PCR amplification, each promoter region was end-labelled with ^32^P-γ ATP. Purified RamA (200 nM) and the different labelled DNA probes (2 nM) were incubated on ice. All reactions were performed on ice prior to electrophoresis on 7.5% native gel. Lane 1 of each panel indicates the labelled DNA probe only, Lane 2 is the BSA control and Lane 3 contains RamA+DNA. **C:** Transcription *in vitro* assay of different promoters using the purified RamA protein. The test DNA (2 nM- *yrbF*, *ybhT*, *yhbW*, *acrA*, *nfnB*, *lpxO* and *lpxC*) with the control template (*gnd*) were incubated for transcription *in vitro* [^32^P]α-UTP with (+) or without (-) 200 nM purified RamA. Samples were fractionated by polyacrylamide/urea gel electrophoresis prior to drying and exposure to the phosphorimager. Relative fold increase was determined using densitometric analysis as described previously [55], by first normalizing all test transcription levels to the control promoter (*gnd*) prior to comparison to the no protein control. Statistics was done using One way ANOVA (P value < 0.05) where transcription levels were found to be statistically significant in the presence of purified RamA compared to the no protein control.

**Fig 7:**
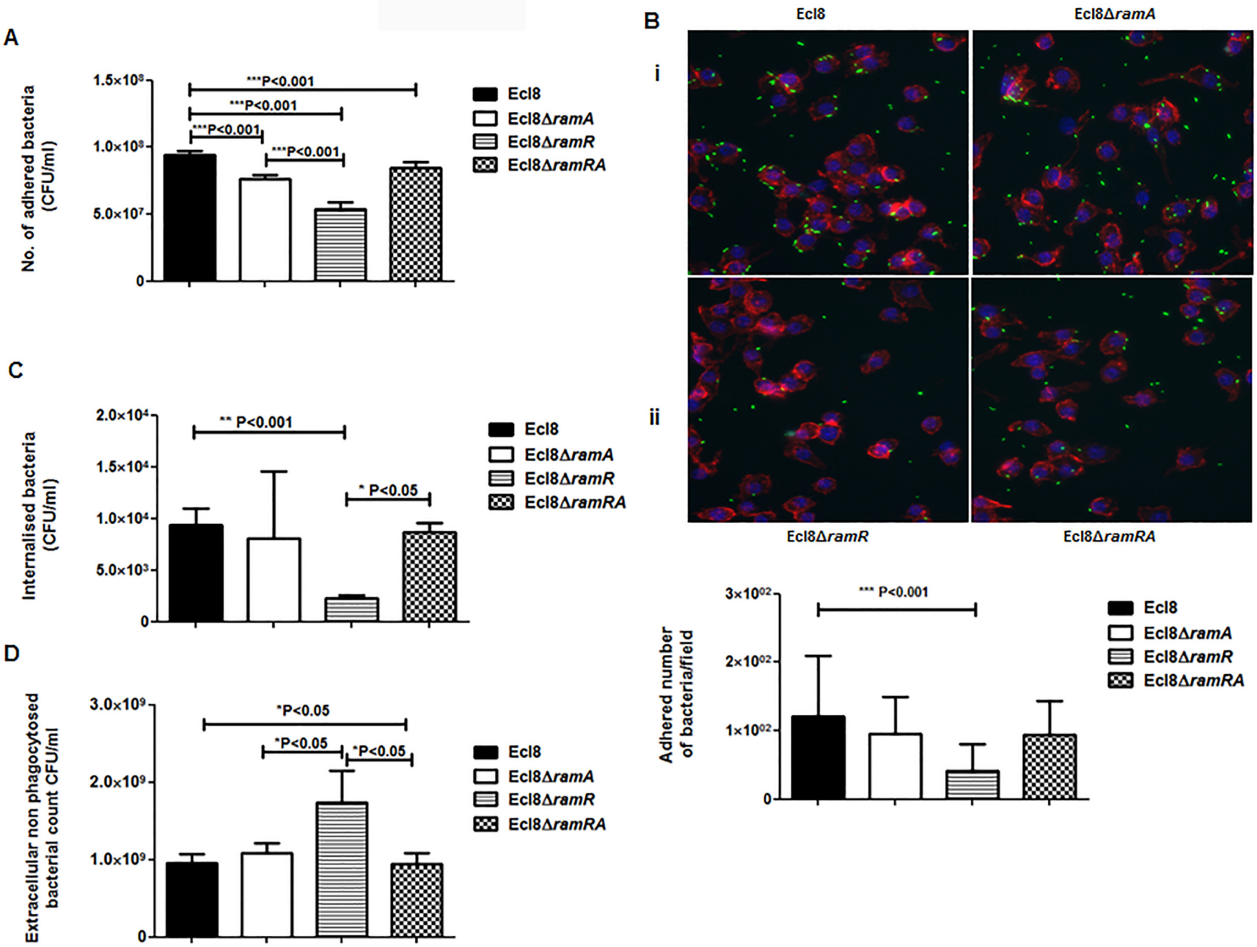
**A: Attachment of *K*. *pneumoniae* Ecl8, Ecl8Δ*ramA*, Ecl8Δ*ramR* or Ecl8Δram*RA* to murine macrophage RAW 264.7 cell line.** One-way ANOVA analyses were performed to demonstrate statistical significance. **B:** Microscopy to assess attachment to RAW 264.7 cell line. (i) Infection of the RAW264.7 cell line was carried out with *K*. *pneumoniae* Ecl8 (WT), Ecl8Δ*ramA*, Ecl8Δ*ramR* or Ecl8Δ*ramRA* transformed with plasmid pRSMgfp. MOI was 1:100 and infections were carried out for 2 hrs. The actin cytoskeleton was stained with Acti stain 555 phalloidin (red) and host cell nuclei were stained with DAPI (blue). Images are representative of 80 fields. (ii) Graph representating mean values are derived from 3 independent experiments. One-way ANOVA analyses (P<0.001) were performed to demonstrate statistical significance. **C:** Internalisation of *K*. *pneumoniae* Ecl8, Ecl8Δ*ramA*, Ecl8Δ*ramR* or Ecl8Δ*ramRA* by RAW 264.7 cells. Bacterial internalisation was assessed by the gentamicin protection assay. One-way ANOVA analyses were performed to demonstrate statistical significance. **D:** Enumeration of the extracellular non-phagocytosed *K*. *pneumoniae* Ecl8, Ecl8Δ*ramA*, Ecl8Δ*ramR* or Ecl8Δ*ramRA*. One-way ANOVA analyses were performed to demonstrate statistical significance.

The authors confirm that these changes do not alter their findings. The authors have provided raw, uncropped blots for Fig 4B as Supporting Information.

## Supporting Information

S1 Fig Uncropped blots for Fig 4B. Raw data phosphor image scan for the EMSA analyses on the *ybhT* and *yrbF* promoters. The experimental setup was as described previously in Fig 4. Briefly, Electrophoretic Mobility Shift Assay (EMSA) using purified RamA protein. Following PCR amplification, each promoter region was end-labelled with ^32^P-γ ATP. Purified RamA (200 nM) and the labelled DNA probes (2 nM) were incubated on ice. All reactions were performed on ice prior to electrophoresis on 7.5% native gel. Lane 1 of each panel indicates the labelled DNA probe only, Lane 2 is the BSA control and Lane 3 contains RamA+DNA. The dried gel was scanned after overnight exposure to the phosphor screen under default settings on the phosphorimager Typhoon FLA7000IP (GE Healthcare).
(PDF)
