# Supplementary figures and images for: Elucidation of the RamA Regulon in Klebsiella pneumoniae Reveals a Role in LPS Regulation
Source: PLoS Pathog. 2015 Jan 29;11(1):e1004627. doi: 10.1371/journal.ppat.1004627 (PMC4310594; doi:10.1371/journal.ppat.1004627)

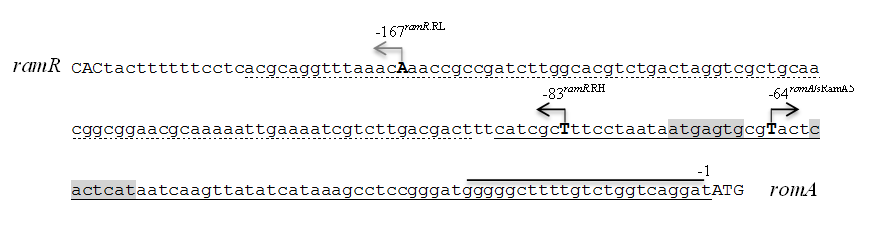

Supplement: S1 Fig — Capitalized triplets are start codons of either ramR or romA. Bold capital letters indicate the primary and secondary TSS sites for ramR or sRamA5 and romA. The primary and secondary TSSs of ramR are indicated with a black and grey arrow respectively. The shaded segments are the inverted repeat (IR) sequences recognized by the RamR protein. The sequences indicated by the dotted underlined fragment (RL) and the single underlined fragment (RH) were used in eGFP analysis. The numbering system is based on the “t” prior to romA’s start codon ATG as the -1. (TIF) [file ppat.1004627.s001.tif]

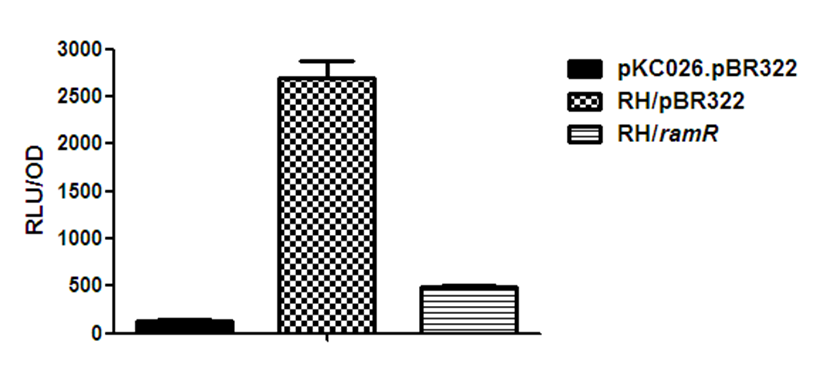

Supplement: S2 Fig — Where indicated DH5α contained both the pKC026 (containing either the RH fragments) and pBR322ramR plasmid. DH5α haboring both pKC026 and pBR322 is taken as a negative control and the RH fragment containing pKC026 with pBR322 only taken as the baseline control. (TIF) [file ppat.1004627.s002.tif]

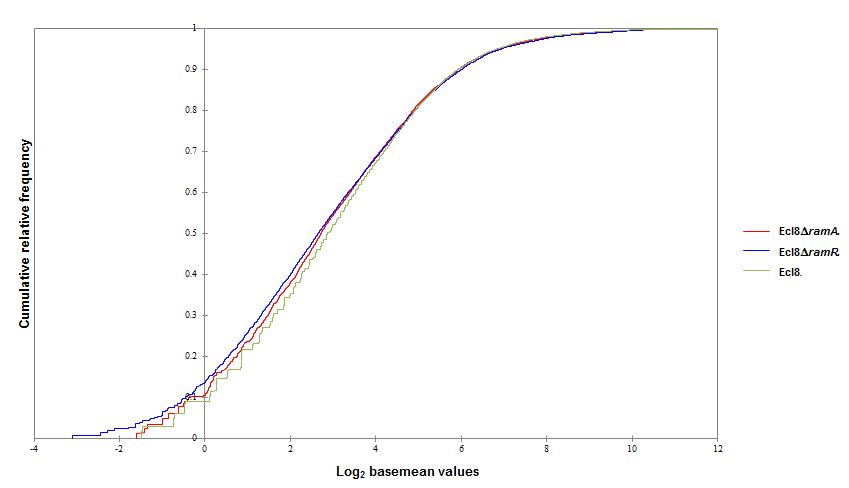

Supplement: S3 Fig — The plots show the sample distribution of log2-transformed basemean values from the RNAseq experiment for Ecl8, Ecl8ΔramA and Ecl8ΔramR. The D value (the largest vertical distance between two curves) between Ecl8 and Ecl8ΔramA is 0.046; the one between Ecl8 and Ecl8ΔramR is 0.067, and the one between Ecl8ΔramA and Ecl8ΔramR is 0.036. All of the distributions are significantly different from each other (p < 0.001). (TIF) [file ppat.1004627.s003.tif]

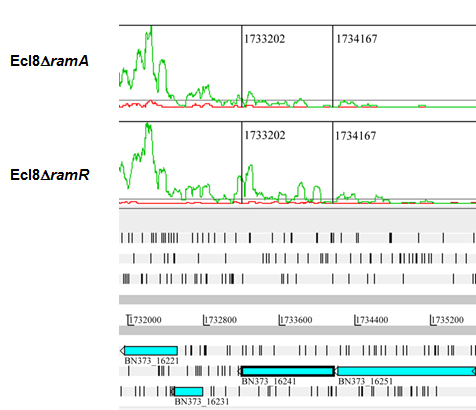

Supplement: S4 Fig — The coverage plot is visualized with Artemis Genome Browser using the Ecl8 genome as a reference. Window size is set at 3. One representative coverage plot of Ecl8ΔramA and Ecl8ΔramR is shown here. The borders of the coding region of BN373_16241 are marked by vertical black bars in the coverage plot. The green and red curves within the borders represent the antisense and sense transcription of BN373_16241 respectively. (TIF) [file ppat.1004627.s004.tif]

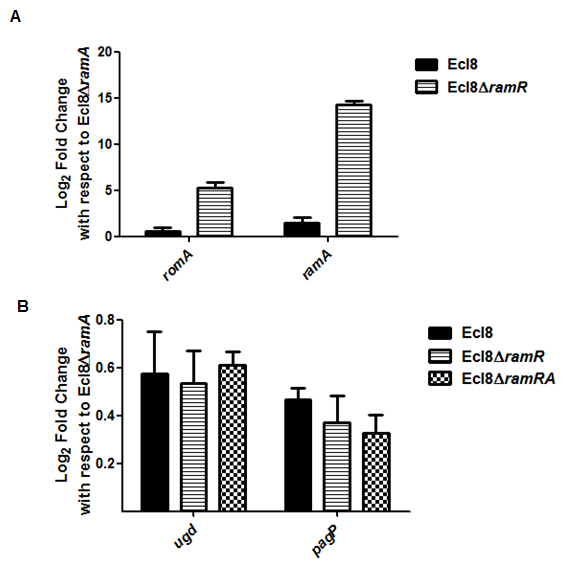

Supplement: S5 Fig — B: Gene expression of ugd, pagP genes in different K. pneumoniae strains. All qPCR experiments were performed as outlined in materials and methods. Expression levels were normalized to 16S levels, and fold change values were generated by calibrating against Ecl8∆ramA. All data is a mean of 3 experiments. (TIF) [file ppat.1004627.s005.tif]

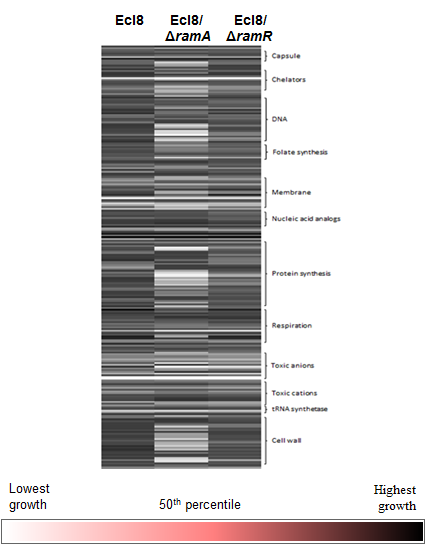

Supplement: S6 Fig — Biolog analyses of the wild type K. pneumoniae Ecl8, Ecl8ΔramA and K. pneumoniae Ecl8ΔramR using PM1–20 plates. (TIF) [file ppat.1004627.s006.tif]
