# Supplementary material for: Elucidation of the RamA Regulon in Klebsiella pneumoniae Reveals a Role in LPS Regulation
Source: PLoS Pathog. 2015 Jan 29;11(1):e1004627. doi: 10.1371/journal.ppat.1004627 (PMC4310594; doi:10.1371/journal.ppat.1004627)
Supplement: S1 Text — (DOCX) [file ppat.1004627.s007.docx]

**Supporting Text S1: Supplementary Materials and Methods**

**RNA-seq Read Mapping and Data Analysis**

Illumina reads per sample were mapped using the Burrows Wheeler Alignment protocol [1] to the genome of Ecl8 (EMBL accession numbers for chromosome and plasmid are HF536482 and HF536483 [2]). The raw data of mapped reads and other QC parameters per sample are summarised in Table S1. The RNA-seq data was submitted to the European Nucleotide Archive with accession code **ERP001994** and ArrayExpress acc. no. **E-ERAD-122**. The mapped reads were firstly aligned against the Ecl8 genome followed by the removal of duplicate reads and correction to allow the directional fidelity of the data to be maintained [3]. An in-house pipeline is implemented at the WTSI for this purpose. The output files includes a matrix of readcounts and RPKM values per sample in both sense and antisense directions for genes as well as automatic 50bp +/- trimmed intergenic features created in the +strand in addition to coverage plots of number of mapped reads per base of sequence. A final matrix of readcounts was prepared for all samples and used as the input file in the R package DESeq, which implements negative binomial distribution statistics for RNA-seq data [4], choosing to estimate dispersions in a per-condition mode [4]. We chose a p-value cut off of 0.07 with a false discovery rate test included (padj values) to assess differentially expressed genes between strains, where genes with less or greater than two-fold changes in basemean values were considered as differentially expressed (Table S1).

A direct pairwise comparison of gene expression levels was also performed using basemean values after normalization to the library size for each sample. The K-S test indicates that the distribution of reads were found to be significantly different (K-S Test, p < 0.001) between the pairwise comparisons of Ecl8Δ*ramA* and Ecl8, Ecl8Δ*ramR* and Ecl8, and Ecl8Δ*ramA* and Ecl8Δ*ramR* (Fig. S3) [5].

**Phenotypic Microarray Analyses**

The phenotypic profile of Ecl8∆*ramR* was compared to that of Ecl8∆*ramA* to determine growth differences in varying substrates using Omnilog phenotype microarrays PM 11-20 (Biolog, California, USA) [6]. Biolog Phenotype Microarray (PM) tests are performed in 96-well microplates containing different nutrients or inhibitors in which cell respiration is measured with a redox indicator. The assay uses a tetrazolium dye, where the reduction of this dye leads to irreversible colour changes in the wells that can be quantified and monitored [7], with respiration serving as a surrogate measure for growth. The Biolog microarrays were set up according to a protocol specified previously [6]. Briefly, bacteria were grown on LB agar overnight at 37 ^o^C. Colonies were picked with a sterile cotton swab and re-suspended in 10 ml IF-0a (Biolog) and adjusted to a transmittance value of 85% using a Biolog transmittance meter. An aliquot of 600 µl of this suspension was added to 120 ml of IF-10 and the 96 well microtiter plates were inoculated with 100 µl/well of this final suspension. The plates were incubated for 48 hours in the Omnilog incubator reader. Data analysis was undertaken using a 10000 AU cut-off and analyzed using the Kinetic Plot and Parametric modules of the Omnilog Phenotype Microarray software suite.

**qPCR using Locked Nucleic Acid (LNA) probe**

In order to assess the levels of sRamA5, primers (FsRamA5 and RsRamA5) and LNA probe for sRamA5 were designed and synthesised by Integrated DNA Technology. The LNA probe has a 5’-labelled FAM as reporter and 3’-labelled Black Hole Quencher (5’ /56-FAM/AGT TAT ATC A+TA AAG CCT CC+G G+GA T/3IABkFQ/ 3’). qPCR with the LNA probe was performed as described in the above section. All sequences of the primers can be found in Supplementary Table S4.

**RNA EMSA**

In order to determine the interaction between sRamA5 and RamR, RNA EMSA was performed as described in [8]. Briefly, the RNA template was produced from DNA templates containing the T7 promoter sequence (TAATACGACTCACTATAGGGctcacgcaggtttaaacaaaccgccgatcttggcacgtctgactaggtcgctgcaacggcggaacgcaaaaattgaaaatcgtcttgacgactttcatcgctttcctaataatgagtgcgtactcactcataatcaagttatatcataaagcctccgggatgggggcttttgtc, T7 promoter sequence is capitalized) with MEGAshortscript T7 kit (Invitrogen, UK) using [α^32^P]-UTP as per the manufacturer’s instructions. Labelled RNA was dissolved in buffer containing 20 mM HEPES-KOH (pH 7.4), 80 mM NaCl, 10 mM KCl, 2.5 mM MgCl_2_, heat-denatured (2 min at 85 °C) and subsequently placed on ice. The RNA and recombinant RamR were mixed in buffer containing 20 mM HEPES-KOH (pH 7.4), 80 mM NaCl, 10 mM KCl, 2.5 mM MgCl_2_, 2 mM DTT, 10 % glycerol, 5 μg tRNA and 5 U of ribonuclease inhibitor (RNasin, Fermentas, UK), and incubated on ice for 1 hour prior to electrophoresis at 100 V on a 8 % native polyacrylamide gel in 1 × TBE buffer.

**Lipid A Analyses**

Lipid A was extracted as described previously with minor modifications [9,10]. Briefly, bacteria were pelleted from 5-ml overnight cultures grown in LB. The cell pellet was washed once with PBS and resuspended in 400 µl of isobutyric acid-1M ammonium hydroxide (5:3 [vol/vol]). Samples were incubated in a screw cap test tube at 100 °C for 2 h with some agitation. Samples were then cooled in ice water and centrifuged (2000xg for 15 mins). The supernatant was transferred to a new tube and 1 vol of water was added. Samples were dried in a Speed-Vac. Dried pellets were washed twice with methanol and the lipid A component was solubilized in 100-200 µl of chloroform-methanol-water (3:1.5:0.25 [vol/vol/vol]). Analyses were performed on a Bruker Autoflex II MALDI-TOF mass spectrophotometer (Bruker Daltonics Inc.) in negative reflective mode with delayed extraction. The settings for sample analyses were as described previously [9]. Data interpretation was based on previous negative-ion spectra analyses for the wild type *Klebsiella pneumoniae* [9].

**Phagocytosis assay using fluorescence microscopy**

RAW cells were seeded on 8 chamber cell culture slides (Millipore EZslide) with 5 × 10^4^ cells per chamber. Infections were carried out as described before with *K. pneumoniae* strains harbouring pRSMgfp+ Cm at MOI of 1:100. After 2 h, cells were washed twice with PBS, prior to being fixed with 3.7 % paraformaldehyde pH 7.4 for 10 mins. Following a further PBS wash for 1 min the cells were made permeable for staining by 0.5 % Triton-X 100 for 5 mins. Actin cytoskeleton was stained with 100 nM Acti stain 555 phalloidin (Cytoskeleton Inc., USA) for 30 mins, followed by staining of DNA with 300 nM DAPI (Invitrogen, UK) for 5 mins. Slide was washed twice in PBS for 5 mins each. Finally, coverslips were mounted using Prolong Gold (Molecular Probes) and analysed with a fluorescent microscope (Leica DM5500B). The number of bacteria in the scanned fields was calculated using a code programme written in Matlab.

**References**

1. Li H, Durbin R (2009) Fast and accurate short read alignment with Burrows-Wheeler transform. Bioinformatics 25: 1754-1760.

2. Fookes M, Yu J, De Majumdar S, Thomson N, Schneiders T (2013) Genome Sequence of *Klebsiella pneumoniae* Ecl8, a Reference Strain for Targeted Genetic Manipulation. Genome Announc 1.

3. Croucher NJ, Fookes MC, Perkins TT, Turner DJ, Marguerat SB, et al. (2009) A simple method for directional transcriptome sequencing using Illumina technology. Nucleic Acids Res 37: e148.

4. Anders S, Huber W (2010) Differential expression analysis for sequence count data. Genome Biol 11: R106.

5. Yoder-Himes DR, Chain PS, Zhu Y, Wurtzel O, Rubin EM, et al. (2009) Mapping the Burkholderia cenocepacia niche response via high-throughput sequencing. Proc Natl Acad Sci U S A 106: 3976-3981.

6. O'Regan E, Quinn T, Pages JM, McCusker M, Piddock L, et al. (2009) Multiple regulatory pathways associated with high-level ciprofloxacin and multidrug resistance in *Salmonella enterica* serovar enteritidis: involvement of RamA and other global regulators. Antimicrob Agents Chemother 53: 1080-1087.

7. Bochner BR, Gadzinski P, Panomitros E (2001) Phenotype microarrays for high-throughput phenotypic testing and assay of gene function. Genome Res 11: 1246-1255.

8. Chinni SV, Raabe CA, Zakaria R, Randau G, Hoe CH, et al. (2010) Experimental identification and characterization of 97 novel npcRNA candidates in *Salmonella enterica* serovar Typhi. Nucleic Acids Res 38: 5893-5908.

9. Llobet E, Campos MA, Gimenez P, Moranta D, Bengoechea JA (2011) Analysis of the networks controlling the antimicrobial-peptide-dependent induction of *Klebsiella pneumoniae* virulence factors. Infect Immun 79: 3718-3732.

10. Perez-Gutierrez C, Llobet E, Llompart CM, Reines M, Bengoechea JA (2010) Role of lipid A acylation in *Yersinia enterocolitica* virulence. Infect Immun 78: 2768-2781.
