# Supplementary figures and images for: Correction: Elucidation of the RamA Regulon in Klebsiella pneumoniae Reveals a Role in LPS Regulation
Source: PLoS Pathog. 2016 May 11;12(5):e1005649. doi: 10.1371/journal.ppat.1005649 (PMC4864251; doi:10.1371/journal.ppat.1005649)

# EMSA Analyses of RamA-Regulated promoters *yrbF* and *ybhT*

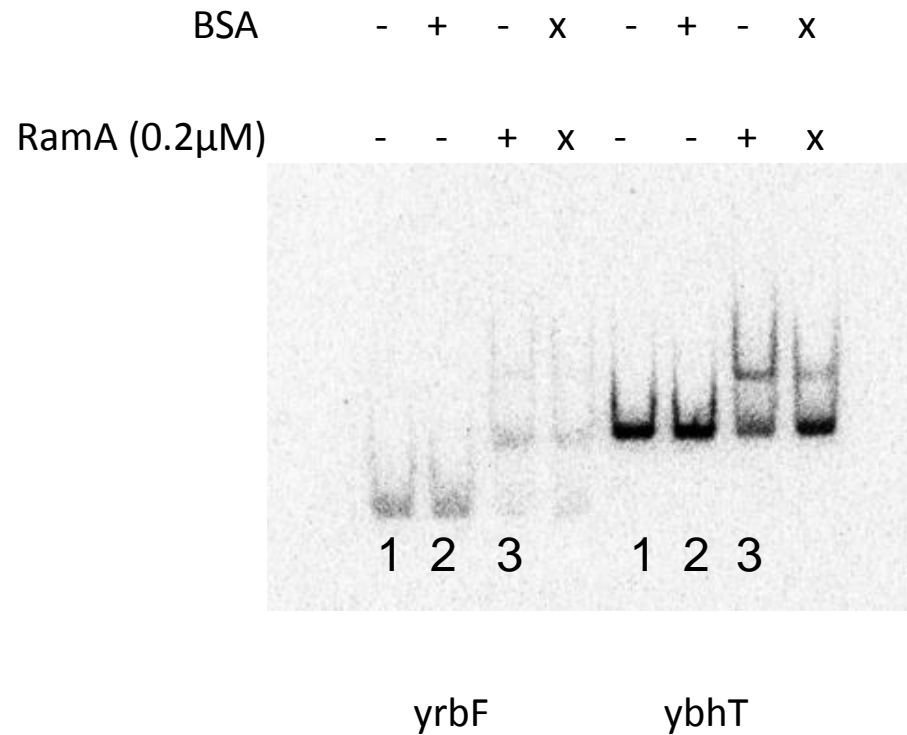

2013-2-22

Supplement: S1 Fig — Raw data phosphor image scan for the EMSA analyses on the ybhT and yrbF promoters. The experimental setup was as described previously in Fig 4. Briefly, Electrophoretic Mobility Shift Assay (EMSA) using purified RamA protein. Following PCR amplification, each promoter region was end-labelled with 32P-γ ATP. Purified RamA (200 nM) and the labelled DNA probes (2 nM) were incubated on ice. All reactions were performed on ice prior to electrophoresis on 7.5% native gel. Lane 1 of each panel indicates the labelled DNA probe only, Lane 2 is the BSA control and Lane 3 contains RamA+DNA. The dried gel was scanned after overnight exposure to the phosphor screen under default settings on the phosphorimager Typhoon FLA7000IP (GE Healthcare). (PDF) [file ppat.1005649.s001.pdf]
